# Physical Activity Induces Nucleus Accumbens Genes Expression Changes Preventing Chronic Pain Susceptibility Promoted by High-Fat Diet and Sedentary Behavior in Mice

**DOI:** 10.3389/fnins.2019.01453

**Published:** 2020-01-22

**Authors:** Arthur Freitas Brandão, Ivan José Magayewski Bonet, Marco Pagliusi, Gabriel Gerardini Zanetti, Nam Pho, Cláudia Herrera Tambeli, Carlos Amilcar Parada, André Schwambach Vieira, Cesar Renato Sartori

**Affiliations:** ^1^Department of Structural and Functional Biology, Institute of Biology, University of Campinas, Campinas, Brazil; ^2^eScience Institute, University of Washington, Seattle, WA, United States

**Keywords:** chronic pain, nucleus accumbens, transcriptome, physical activity, high-fat diet, neuroplasticity

## Abstract

Recent findings from rodent studies suggest that high-fat diet (HFD) increases hyperalgesia independent of obesity status. Furthermore, weight loss interventions such as voluntary physical activity (PA) for adults with obesity or overweight was reported to promote pain reduction in humans with chronic pain. However, regardless of obesity status, it is not known whether HFD intake and sedentary (SED) behavior is underlies chronic pain susceptibility. Moreover, differential gene expression in the nucleus accumbens (NAc) plays a crucial role in chronic pain susceptibility. Thus, the present study used an adapted model of the inflammatory prostaglandin E2 (PGE2)-induced persistent hyperalgesia short-term (PH-ST) protocol for mice, an HFD, and a voluntary PA paradigm to test these hypotheses. Therefore, we performed an analysis of differential gene expression using a transcriptome approach of the NAc. We also applied a gene ontology enrichment tools to identify biological processes associated with chronic pain susceptibility and to investigate the interaction between the factors studied: diet (standard diet vs. HFD), physical activity behavior (SED vs. PA) and PH-ST (PGE vs. saline). Our results demonstrated that HFD intake and sedentary behavior promoted chronic pain susceptibility, which in turn was prevented by voluntary physical activity, even when the animals were fed an HFD. The transcriptome of the NAc found 2,204 differential expression genes and gene ontology enrichment analysis revealed 41 biologic processes implicated in chronic pain susceptibility. Taking these biological processes together, our results suggest that genes related to metabolic and mitochondria stress were up-regulated in the chronic pain susceptibility group (SED-HFD-PGE), whereas genes related to neuroplasticity were up-regulated in the non-chronic pain susceptibility group (PA-HFD-PGE). These findings provide pieces of evidence that HFD intake and sedentary behavior provoked gene expression changes in the NAc related to promotion of chronic pain susceptibility, whereas voluntary physical activity provoked gene expression changes in the NAc related to prevention of chronic pain susceptibility. Finally, our findings confirmed previous literature supporting the crucial role of voluntary physical activity to prevent chronic pain and suggest that low levels of voluntary physical activity would be helpful and highly recommended as a complementary treatment for those with chronic pain.

## Introduction

Obesity and chronic pain are two highly prevalent conditions associated with a modern lifestyle in both developed and developing countries. A meta-analysis study from 1975 to 2016 showed an increase in overweight and obesity prevalence worldwide and the authors argued that an unhealthy nutritional transition (i.e., childhood to adulthood) and an increased intake of nutrient-poor with high energy-dense foods (e.g., fat and sugar) lead to weight gain (Abarca-Gómez et al., 2017). At the same time, epidemiological studies indicate an increased number of humans with chronic pain in recent years (Torrance et al., 2006; Van Hecke et al., 2013, 2014). In the United States, the economic impact of chronic pain is estimated to be a cost between $560 and $635 billion annually (Gaskin and Richard, 2012). Furthermore, pain and chronic pain conditions have major impacts on an individual’s work, personal and social life (Breivik et al., 2013; Mayer et al., 2019).

A cross-sectional study from low- and middle-income countries showed that sedentary behavior is strongly related to obesity and chronic pain and suggested that interventions focusing on reducing sedentary behavior should be considered for these chronic conditions (Koyanagi et al., 2018). A recent study in adults with obesity or overweight demonstrated that weight loss interventions such as voluntary physical activity promoted significant pain reduction (Cooper L. et al., 2018), adding to the known effects and growing literature relating the benefits of increased physical activity for treatment of chronic pain (Geneen et al., 2017; Lima et al., 2017a, b) and obesity (Paley and Johnson, 2016). However, studies investigating the interactions between high-fat diet (HFD), sedentary behavior, voluntary physical activity in chronic pain susceptibility are limited.

It is well-known that the central nervous system regulates energy homeostasis and obesity (Gautron et al., 2015), and several studies suggest that brain neuroplasticity underlies chronic pain (Apkarian et al., 2009; Mansour et al., 2014; Doan et al., 2015; Descalzi et al., 2017) and obesity (Matikainen-Ankney and Kravitz, 2018; Mattson et al., 2018). The nucleus accumbens (NAc) has been investigated as having a critical role in modulating chronic pain in humans and animal models (Martikainen et al., 2015; Ren et al., 2015; Benarroch, 2016; Schwartz et al., 2017). Transcriptomic studies have revealed novel insights related to chronic pain including differential gene expression and disrupted biological processes (Starobova et al., 2018). For instance, to investigate migraine-associated hyperalgesia, a recent transcriptome study of mouse NAc and trigeminal ganglia showed gene network dysregulation and biological processes alteration in both areas (Jeong et al., 2018). Through gene ontology enrichment tools, the authors identified crucial up-regulated biological processes in both areas related to hyperalgesia, such as amino acid transmembrane, anion transmembrane transport, and amino acid transport (Jeong et al., 2018). Another chronic pain transcriptome study of mouse NAc, medial prefrontal cortex (mPFC), and periaqueductal gray (PAG) showed that gene expression changes in these brain areas were strongly related to other comorbidities, including depression and stress (Descalzi et al., 2017).

Additionally, Jayaraman et al. (2014) demonstrated that cultured glial cells generated from high-fat-fed animals exhibit reduced survival, poorer neurite outgrowth, and evidence of nerve damage in the peripheral nervous system. Although these alterations have been identified from primary neurons co-cultured with glial cultures, investigation of biological processes related to metabolic and mitochondrial stress in the NAc associated with HFD, chronic pain susceptibility, and voluntary physical activity remain elusive. Thus, further understanding of the complex biological processes that may underlie chronic pain susceptibility, including if HFD and sedentary behavior induce chronic pain, and if voluntary physical activity can prevent it, is needed. And, further investigating potential alterations in the transcriptome of the NAc can shed light and reveal novel interesting insights.

Therefore, the first objective of the current study was to investigate whether HFD and sedentary behavior promote chronic pain susceptibility and whether voluntary physical activity can prevent it. Our second objective was to describe the differential gene expression in the NAc related to chronic pain susceptibility under conditions of HFD and sedentary behavior and, voluntary physical activity. Finally, we also applied gene ontology enrichment tools to describe the biological processes related to chronic pain susceptibility promoted by an HFD and sedentary behavior and prevented by voluntary PA.

## Materials and Methods

### Animal

To test our hypothesis, eighty male C57BL/6JUnib mice at the age of 4 weeks were used from the Multidisciplinary Center for Biological Investigation on Laboratory Animal Science of the University of Campinas (CEMIB – UNICAMP). The mice were housed in a temperature-controlled room (21 ± 1°C) on a 12 h light/dark cycle (lights on at 7 AM) with *ad libitum* access to standard chow and water. Before the beginning of the experiment, all mice were acclimated to the vivarium in collective cages (five mice per cage) for 2 weeks. At 6 weeks old, the mice were randomly placed in individual cages through the end of the experiment (at 18 weeks old). All procedures were reviewed and approved in accordance with the Brazilian federal law on animal experimentation (n° 11.794 October 08, 2008) and by the University of Campinas Animal Ethics Committee (CEUA-UNICAMP) under protocol number 4243-1. All efforts were made to reduce the number of animals used and minimize animal suffering.

### Experimental Design

**FIGURE 1:**
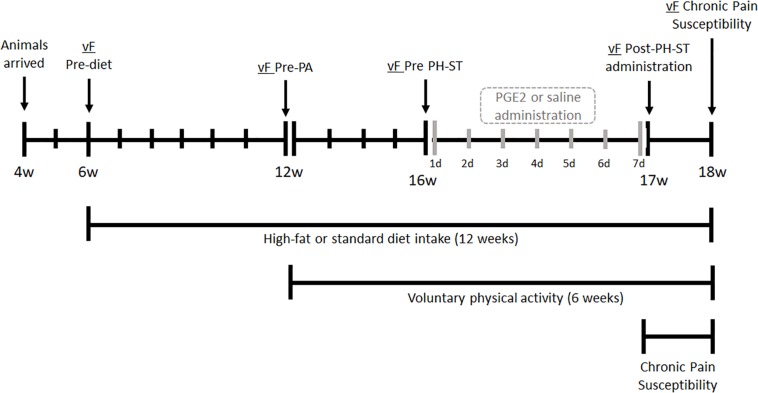
Experimental design. At the age of 4 weeks, mice arrived were acclimated to the vivarium for 2 weeks. At 6 weeks old, mice underwent the vF Pre-diet test, then were divided into two groups and assigned to a standard or high-fat diet. At 12 weeks old, the vF Pre-PA test was completed, then assigned diet groups were subdivided into behavior groups: physical activity (PA-SD and PA-HFD) had free running wheel access for voluntary physical activity or sedentary (SED-SD and SED-HFD) which remained in a standard cage for 6 weeks. At 16 weeks old, each of the four groups were submitted to the vF Pre-PH-ST test before administration of PGE2 (PH-ST protocol) or saline. At 17 weeks old, all eight groups underwent the vF Post-PH-ST administration test. At 18 weeks old, all eight groups completed the vF chronic pain susceptibility test and were euthanized. vF, von Frey; PA, physical activity; PH-ST, persistent hyperalgesia short-term protocol; PGE2, prostaglandin E2; w, age in weeks old, d, days.

At 6 weeks old, mice were randomly separated into two groups: one was fed a standard diet (SD; *n* = 40) and the other was fed a HFD (*n* = 40) for 12 weeks (Figure 1). At 12 weeks old, or after 6 weeks feeding on assigned diet, the SD and HFD groups remained on assigned diet and were randomly subdivided into four groups: (i) sedentary/standard diet (SED-SD; *n* = 20) and, (ii) sedentary/high-fat diet (SED-HFD; *n* = 20) groups, which were placed in standard cages without running wheel (RW) access; (iii) physically active/standard diet (PA-SD; *n* = 20) and (iv) physically active/high-fat diet (PA-HFD; *n* = 20) groups, which were placed in cages with free access to a RW for 6 weeks. At 16 weeks old, each group was subdivided into two sub-groups, one of those sub-groups was submitted to prostaglandin E2 (PGE2)-induced persistent hyperalgesia with a short-term (PH-ST) protocol and the other group was submitted to a saline administration as a PGE2 control group (details below, see section “Prostaglandin E2-Induced Persistent Hyperalgesia Short-Term Protocol”). Thus, the experimental design had eight experimental groups divided according to Table 1. Moreover, eleven mice were removed from the analysis because they did not meet the inclusion criteria described in section “Delta Mechanical Nociceptive Threshold.”

**TABLE 1:** Description of experimental groups.

Groups	Description	*n1, n2, n3*
SED-SD-SAL	Sedentary mice fed a standard diet receiving saline	10-8-5
SED-SD-PGE	Sedentary mice fed a standard diet receiving PGE	10-9-5
PA-SD-SAL	Physically active mice fed a standard diet receiving saline	10-8-5
PA-SD-PGE	Physically active mice fed a standard diet receiving PGE	10-9-5
SED-HFD-SAL	Sedentary mice fed a high-fat diet receiving saline	10-8-5
SED-HFD-PGE	Sedentary mice fed a high-fat diet receiving PGE	10-9-5
PA-HFD-SAL	Physically active mice fed a high-fat diet receiving saline	10-9-5
PA-HFD-PGE	Physically active mice fed a high-fat diet receiving PGE	10-9-5

*SD, standard diet; HFD, high-fat diet; SED, sedentary; PA, physically active; PGE, prostaglandin E2; and SAL, saline. N, number of animals of each stage: *n1*, initial mice/group; *n2*, mice/group behavioral analysis; *n3*, mice/group transcriptome analysis.*

Furthermore, we assessed of mechanical nociceptive threshold through an electronic von-Frey (vF) test at five timepoints. The first vF test was performed prior to diet exposure (vF Pre-diet) at 6 weeks old. The second vF test was performed at 12 weeks old prior to RW access for voluntary physical activity (vF Pre-PA). The third vF test was performed at 16 weeks old and was measured 1 h before the PGE2-induced PH-ST protocol or saline administration (vF Pre-PH-ST). The fourth vF test was performed at 17 weeks old and was measured 1 day after the end of PGE2-induced PH-ST protocol or saline administration (vF Post-PGE2 administration). The fifth vF test was performed 7 days after the end of the PH-ST protocol, at 18 weeks old (Figure 1). During the chronic pain susceptibility period, from 17 until 18 weeks old, the mice were not manipulated.

### Body Mass, Caloric Intake, and Diets

Body mass and food intake were recorded weekly, always on the same weekday and at the same time of the day (1 PM ± 1 h, light cycle). The SD (3.080 kcal/g) was composed by 11.7% kcal/g from lipid, 28.5% kcal/g from protein and, 59.7% kcal/g from carbohydrate (Nuvilab-Quimtia S/A, Paraná, Brazil) (Table 2). The HFD (5.439 kcal/g) was composed by 58.2% kcal/g from lipid (51.61% from lard and 6.61% kcal/g from soybean oil), 14.9% kcal/g from protein and, 26.8% kcal/g from carbohydrate provided by Laboratory of Nutritional Genomics from the School of Applied Science of the University of Campinas (Cintra et al., 2012).

**TABLE 2:** Distribution of macronutrients and caloric equivalent (kcal/g) of diets.

Macronutrients	Standard diet (kcal %)	High-fat diet (kcal %)
Lipid	11.7	58.2
Protein	28.5	14.9
Carbohydrate	59.7	26.8
TOTAL	3.080 kcal/g	5.439 kcal/g

### Adipose Tissue Dissection

To assess adiposity, epididymal and retroperitoneal adipose tissue was dissected and weighed as described by de Jong et al. (2015). Tissue was snap-frozen in liquid nitrogen and stored at −80°C. Adipose tissue weight (in grams) from each depot was normalized to total body mass (in grams) and is presented as mean percent of total body mass and standard error.

### Prostaglandin E2-Induced Persistent Hyperalgesia Short-Term Protocol

To test chronic pain susceptibility, we employed an adapted model of the inflammatory PGE2-induced persistent hyperalgesia short-term (PH-ST) protocol previously described by Ferreira et al. (1990) and Villarreal et al. (2009). Briefly, the original model showed that 14 successive days of intraplantar injection of PGE2 was sufficient to induce persistent hyperalgesia in rodents for more than 30 days after the last PGE2 injection (Ferreira et al., 1990; Villarreal et al., 2009). Here we used a PGE2-induced PH-ST protocol with only seven successive days of intraplantar injection of PGE2, which is not enough to produce persistent hyperalgesia (Ferreira et al., 1990; Dias et al., 2015). Pharmacological studies revealed the role of non-inflammatory agents during the hyperalgesia induction, such as PKA (protein kinase A), PKCε (protein kinase C isoform ε), AC (adenylyl cyclase) (Sachs et al., 2009; Villarreal et al., 2009), and NF-κB (nuclear factor kappa-B) (Souza et al., 2015). Additionally, HFD results in a low-grade inflammatory state in metabolic tissues (Gregor and Hotamisligil, 2011), thus, using the PGE2-induced PH-ST model avoids the potential sum of peripheral inflammatory agents promoted by an HFD and by the hyperalgesia induction model. PGE2 and saline (SAL) administration was made through a hypodermic 26-gauge needle coupled at a 50 μL Hamilton syringe to administer 18 μL [90 ng] of PGE2 (Sigma–Aldrich^®^, St. Louis, MO, United States) or 18 μL of SAL (NaCl 0.9%) in the intraplantar surface of the mice right hind paw. Finally, all groups received their injection (PGE2 or SAL) daily, for 7 days, at the same time of day (1 PM ± 1 h, light cycle) starting and ending at 16–17 weeks old, respectively.

### Delta Mechanical Nociceptive Threshold

The mechanical nociceptive threshold was obtained through an electronic von-Frey (vF) apparatus (Insight, Ribeirão Preto, Brazil) adapted for mice (Martinov et al., 2013; Deuis et al., 2017). Briefly, the vF test was performed in a quiet, temperature-controlled room (21 ± 1°C) and always at the same time of day (1 PM ± 1 h, light cycle). Precisely, 30 min before the vF test, mice were placed in an acrylic cage (12 × 20 × 17 cm) with wire grid floors for acclimation and experimenters remained in the room for appropriate environmental acclimation between mice and experimenters as suggested by Martinov et al. (2013). To measure the hind paw mechanical nociceptive threshold, the experimenter applied constant pressure on the plantar surface of paws (right and left) until the paw-withdrawal threshold. The experimenter was blinded to PGE2-induced PH-ST protocol and to voluntary physical activity or sedentary behavior variables and partially blinded to diet variable because mice from the HFD group were visibly larger than those from the SD group. The stimuli on the plantar surface were repeated (three-minimum or five-maximum times), not consecutively, until three registrable measurements with equal values or with a difference less than 20% (±2.0 g) were obtained (Martinov et al., 2013). Those mice which did not fulfill this criterion of inclusion (e.g., five-maximum attempts) were excluded from the analysis (11 mice).

The values of mechanical nociceptive threshold were reported by the average of measurements performed at each time point. The data are presented as the delta (Δ) of mechanical nociceptive threshold was calculated by the difference between the average baseline value of mechanical nociceptive threshold (vF Pre PH-ST at 16 weeks old) minus the average value of mechanical nociceptive threshold at each of the other time points. For instance, at the time point “−7,” the values were obtained from the vF Pre-PA – vF Pre PH-ST tests. At the time point “1,” the values were obtained from the vF Pre PH-ST – vF Post PH-ST tests and at the time point “7,” the values were obtained from the vF Pre PH-ST – vF chronic pain susceptibility tests. Finally, the higher the values of delta mechanical nociceptive threshold, the higher the chronic pain susceptibility.

### Voluntary Physical Activity

Voluntary physical activity behavior was evaluated through free access to a RW (Columbus Instruments; Columbus, OH, United States) installed in the home cage. Each RW had a magnetic indicator sensor connected to computer software to record summation of wheel revolutions. Distance traveled was determined by RW interior diameter (9.2 cm) and mean of total daily distance traveled from 12 to 18 weeks old (6 weeks or 42 days in total) was calculated using a script routine in MATLAB^®^ software (R12 version from MathWorks, Natick, MA, United States) and Microsoft Excel^®^.

### Nucleus Accumbens Transcriptome Library Preparation

At 18 weeks old, the mice were euthanized by decapitation according to the guidelines from the American Veterinary Medical Association (2013). The brain was immediately removed, snap-frozen in liquid nitrogen, and stored at −80°C. Bilateral microdissected slices at 60 μm thickness of the NAc were obtained using a cryostat (Leica^®^, Wetzlar, Germany) from 1.96 mm up to 0.62 mm to bregma (Franklin and Paxinos, 1996) and fixed on pre-prepared parafilm microscope slides. The parafilm microscope slices were stained with Cresyl Violet 1% diluted in alcohol 50% (Sigma Aldrich) and with 40° optical microscopy, the NAc was identified and microdissected for transcriptome analysis.

Total RNA extraction of the NAc was obtained using TRIzol^®^ reagent (Invitrogen – Waltham, MA, United States) according to the manufacturer’s instructions. The RNA quality was confirmed by RNA 6000 Nano Assay for Agilent 2100 Bioanalyzer (Agilent Technologies, Stockport, United Kingdom). The RNA was quantified using a NanoVue Plus spectrophotometer (GE Healthcare Life Sciences, Pittsburgh, PA, United States) and a fluorometer (Qbuit^®^, Life Technologies, Carlsbad, CA, United States). The cDNA libraries were performed from 200 ng of extracted RNA using the TruSeq Stranded mRNA poliAAA (Illumina^®^, San Diego, CA, United States) according to manufacturer instructions. We performed 40 samples in two group-balanced lanes with 20 samples in each lane and five samples per group. The cDNA libraries were sequenced processed with an Illumina HiSeq^®^ 2500 in High Output mode at the Macrogen NGS Service (Seoul, South Korea), producing 101-bp paired-end reads. The first sequencing run produced a mean of 19,992,450 reads with a mean of 95.54% bases over Q30 per sample. The second run produced a mean of 18,090,251 reads with a mean of 96.94% bases over Q30 per sample. The sequence reads were aligned to the *Mus musculus* genome (GRCm38) from Ensembl^1^ using STAR 2.7.0 aligner (Dobin et al., 2013). Sequence read archive is accessible under the number PRJNA564336 public repository at https://www.ncbi.nlm.nih.gov/.

### Statistical Analyses

First, we performed the Shapiro–Wilk test to test for normality among the variables body mass, food intake, delta mechanical nociceptive threshold, and distance traveled. For all aforementioned variables, the analysis of variance with repeated measures (ANOVA-RM) was applied. For the adipose tissue variable, considering that voluntary physical activity may interfere on the amount of adipose tissue (epididymal and retroperitoneal) in both groups, a factorial ANOVA was applied. The Newman–Keuls *post hoc* was applied when indicated. We also performed Grubb’s test to check outlier’s existence (*p*-value < 0.05) for all variables described above. Statistical testing was done using STATISTICA^®^10 software (StatSoft, Hamburg, Germany) and the data were presented by the mean and standard error of the mean (SEM) except for the body mass result, which was presented by the mean and standard deviation. Graphics were generated using GraphPad Prism 7 software (GraphPad Software, San Diego, CA, United States).

The interaction factors (diet vs. RW vs. PH-ST) of the differential gene expression analyses were performed using the DESeq2 R package library using a cutoff of an adjusted *p*-value of *p* < 0.05 (Love et al., 2014). A Volcano R package library (Blighe, 2019) was used to visualize the differential gene expression. The R script used is available in the Supplementary Data Sheet S1. Functional annotation clustering was performed using the Database for Annotation, Visualization and Integrated Discovery (DAVID v6.8)^2^ using DAVID EASE (Expression Analysis Systematic Explorer) score (*p* < 0.05), a modified Fisher’s exact test corrected for multiple hypothesis testing using Benjamini–Hochberg false discovery rates (FDR *p* < 0.05). The functional categories investigated included gene ontology (GO) of the biological processes.

## Results

### Body Mass, Caloric Intake, and Adipose Tissue

**FIGURE 2:**
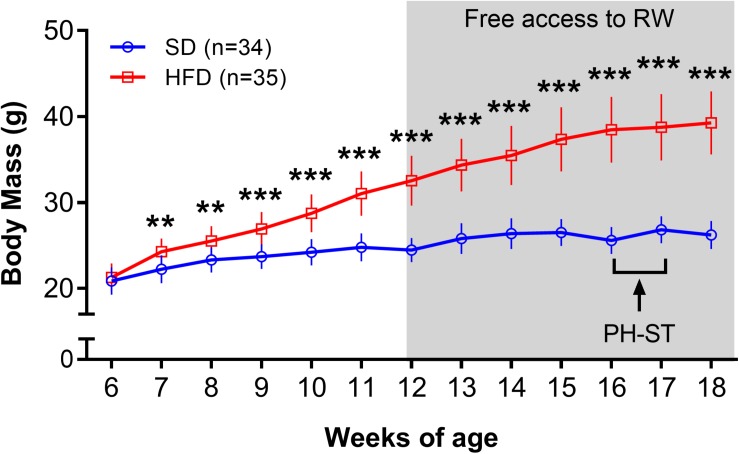
Body mass (grams) throughout the experiment. The ANOVA-RM and the *post hoc* analyses revealed that the body mass was increased in the HFD group when compared to the SD group from 7 to 18 weeks of age. Gray area represents the period a subset of mice had access to a running wheel for voluntary physical activity. Data are mean and standard deviation. SD, standard diet; HFD, high-fat diet; RW, running wheel; PH-ST, persistent hyperalgesia short-term protocol. ** = *p* < 0.01. *** = *p* < 0.001.

The ANOVA-RM revealed a main effect of diet (*F*_(__1_,_67__)_ = 207.75, *p* < 0.001), time (*F*_(__12_,_804__)_ = 745.86, *p* < 0.001) and an interaction effect of diet and time (*F*_(__12_,_804__)_ = 234.67, *p* < 0.001) on the body mass throughout the experiment (Figure 2). *Post hoc* analysis showed from the 7th to 18th weeks of age that the HFD group had significantly higher body mass when compared to an SD group (Week 7: 24.27 ± 0.26 g vs. 22.23 ± 0.27 g, *p* = 0.004; Week 18: 39.26 ± 0.62 vs. 26.24 ± 0.28 g, *p* = 0.0001, respectively).

**FIGURE 3:**
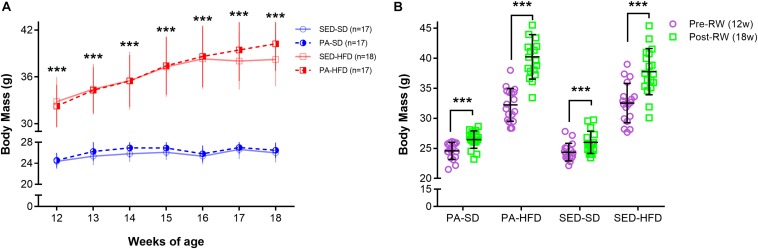
Body mass during the free access to running wheel for voluntary physical activity. In panel **(A)**, the analyses reveal that the SED-HFD and PA-HFD groups had significantly higher body mass when compared to SED-SD and PA-SD groups from 12th until 18th week of age (*** = *p* < 0.001 for both SED-HFD and PA-HFD groups when compare to SED-SD and PA-SD groups). In panel **(B)**, the ANOVA-RM and the *post hoc* analyses reveal that the body mass had a significant increase at the pre-RW and post-RW time point within the voluntary physical activity groups (PA-SD and PA-HFD) and within sedentary groups (SED-SD and SED-HFD) (*** = *p* < 0.001). Data are mean and standard deviation. SED-SD, sedentary standard diet; PA-SD, physical activity standard diet; SED-HFD, sedentary high-fat diet; PA-HFD, physical activity high-fat diet groups.

To assess the effect of voluntary physical activity on body mass, weekly mean body mass during the period of free access to a RW for voluntary physical activity (12–18 weeks) was considered in a three-factor ANOVA-RM model. Results showed a main effect of diet (*F*_(__1_,_65__)_ = 280.11, *p* < 0.001) and time (*F*_(__6_,_390__)_ = 209.65, *p* < 0.001) but did not show a main effect of physical activity (*F*_(__1_,_65__)_ = 0.71, *p* = 0.40) on body mass. Moreover, the test showed an interaction effect of time and physical activity (*F*_(__6_,_390__)_ = 4.27, *p* < 0.001) and an interaction effect of time and diet (*F*_(__1_,_390__)_ = 92.16, *p* < 0.0001) but, the test did not show an interaction effect of diet and physical activity (*F*_(__1_,_65__)_ = 0.17, *p* = 0.89) on body mass. The test also revealed an interaction effect of time, diet and physical activity (*F*_(__6_,_390__)_ = 6.08, *p* < 0.001) on body mass during the period of free access to a RW for voluntary physical activity. *Post hoc* analysis revealed from the 12th to 18th weeks of age that the PA-HFD and SED-HFD groups had significantly higher body mass when compare to the PA-SD and SED-SD groups (Week 12: 32.25 ± 2.72 g and 32.82 ± 3.14 g vs. 24.59 ± 1.41 g, *p* = 0.0001 and 24.37 ± 1.47 g, *p* = 0.0001; Week 18: 40.24 ± 3.71 g and 38.22 ± 3.44 g vs. 26.74 ± 1.45 g, *p* = 0.0001 and 26.0 ± 1.83 g, *p* = 0.0001, respectively) (Figure 3A). *Post hoc* analysis also revealed that mean body mass was a significantly increased at week 18 compared to mean body mass at week 12 (prior to behavioral group assignment) within all groups (PA-SD: 26.47 ± 1.83 g vs. 24.59 ± 1.41 g, *p* = 0.0001; PA-FD: 40.24 ± 3.71 vs. 32.25 ± 2.72, *p* < 0.0001; SED-SD: 26.00 ± 1.83 g vs. 24.37 ± 1.47 g, *p* = 0.0001; SED-HFD 38.22 ± 3.44 g vs. 32.82 ± 3.14 g, *p* = 0.0001) (Figure 3B).

**FIGURE 4:**
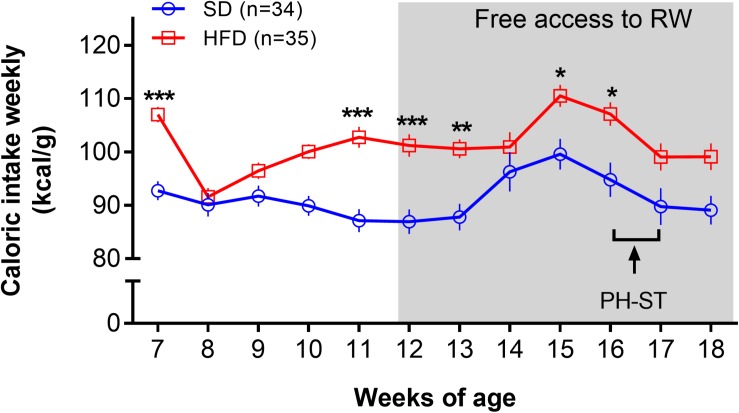
Caloric intake weekly. The ANOVA-RM and the *post hoc* analyses revealed increased caloric intake (kcal/g weekly) in the HFD group when compared to the SD group at the 7th, 11th, 12th, 13th, 15th, and 16th weeks of age. The gray area represents the period a subset of mice had access a running wheel for voluntary physical activity. Data are mean and standard error of the mean. SD, standard diet; HFD, high-fat diet; RW, running wheel; PH-ST, persistent hyperalgesia short-term protocol. * = *p* < 0.05. ** = *p* < 0.01. *** = *p* < 0.001.

The ANOVA-RM test revealed a main effect of diet (*F*_(__1_,_67__)_ = 14.51, *p* < 0.001), time (*F*_(__11_,_737__)_ = 13.83, *p* < 0.001), and an interaction effect of diet and time (*F*_(__11_,_737__)_ = 4.22, *p* < 0.001) on caloric intake. *Post hoc* analysis showed that the HFD group had significantly greater caloric intake when compared to SD group at the 7th (106.98 ± 1.46 kcal/g vs. 92.72 ± 1.79 kcal/g, *p* = 0.001), 11th (102.74 ± 1.97 kcal/g vs. 87.11 ± 2.17 kcal/g, *p* = 0.0005), 12th (101.19 ± 2.11 kcal/g vs. 86.92 ± 2.32 kcal/g, *p* = 0.003), 13th (100.56 ± 1;80 kcal/g vs. 87.77 ± 2.5 kcal/g, *p* = 0.012), 15th (100.52 ± 2.07 kcal/g vs. 99.59 ± 2.88 kcal/g, *p* = 0.029) and 16th (107.11 ± 2.21 kcal/g vs. 94.78 ± 3.20 kcal/g, *p* = 0.013) weeks of age old (Figure 4).

**FIGURE 5:**
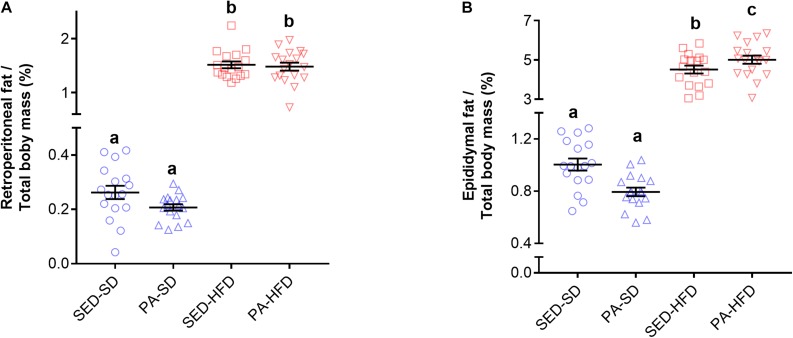
Retroperitoneal and epididymal adipose tissue. Factorial ANOVA and *post hoc* analyses revealed that the both retroperitoneal **(A)** and epididymal **(B)** adipose tissue mass was greater in the SED-HFD and PA-HFD groups when compared to the SED-SD and PA-SD groups and that the epididymal adipose tissue mass was greater in the PA-HFD group when compare to SED-HFD. Data are mean and standard error of the mean. SED-SD, sedentary standard diet group; PA-SD, physical activity standard diet group; SED-HFD, sedentary high-fat diet group; and PA-HFD, physical activity high-fat diet group. *n* = 17–18 group. a vs. b, a vs. c, *p* = 0.0001; b vs. c. *p* = 0.021.

The retroperitoneal and epididymal adipose tissues mass was greater in the HFD groups compared to SD groups. Factorial ANOVA revealed a main effect of diet (*F*_(__1_,_65__)_ = 617.15, *p* < 0.001) on retroperitoneal adipose tissue, but there was no main effect of physical activity (*F*_(__1_,_65__)_ = 0.75, *p* = 0.38) nor an interaction effect of diet and physical activity (*F*_(__1_,_65__)_ = 0.45, *p* = 0.83) on retroperitoneal adipose tissue. *Post hoc* analysis revealed retroperitoneal adipose tissue mass was significantly greater in the HFD groups (PA-HFD 1.48 ± 0.07 g and SED-HFD 1.51 ± 0.06 g) when compared to SD groups (PA-SD 0.20 ± 0.01 g and SED-SD 0.26 ± 0.02 g, *p* < 0.0001) (Figure 5A). For epididymal adipose tissue mass, factorial ANOVA also revealed a main effect of diet (*F*_(__1_,_65__)_ = 674.44, *p* < 0.001) but there was no main effect of physical activity (*F*_(__1_,_65__)_ = 0.92, *p* = 0.33). The test revealed a interaction effect of diet and physical activity (*F*_(__1_,_65__)_ = 5.64, *p* = 0.02). *Post hoc* analysis revealed epididymal adipose tissue was significantly greater in the SED-HFD (4.51 ± 0.82 g) and PA-HFD (5.0 ± 0.87 g) groups when compared to SED-SD (1.00 ± 0.18 g, *p* < 0.001) and PA-SD (0.79 ± 0.13 g, *p* < 0.001) groups (Figure 5B). *Post hoc* analysis also revealed epididymal adipose tissue of the PA-HFD (5.0 ± 0.87 g) group was significantly higher than the SED-HFD group (4.51 ± 0.82 g, *p* = 0.021).

### Voluntary Physical Activity

**FIGURE 6:**
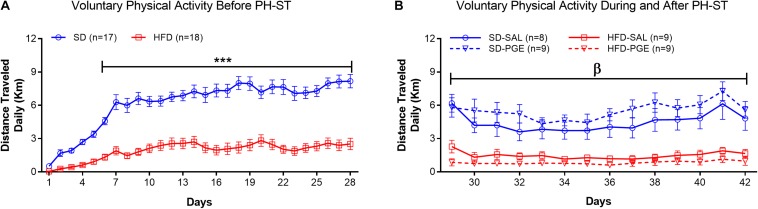
Voluntary physical activity measured by the distance traveled daily, before, during and after the PH-ST protocol. In panel **(A)**, the ANOVA-RM and *post hoc* analyses showed that the SD group had greater distance traveled when compared to the HFD group before the PH-ST protocol (during 12–16 weeks of age). In panel **(B)**, the ANOVA-RM and *post hoc* analyses showed that the SD-SAL and SD-PGE groups had greater distance traveled when compared HFD-SAL and PGE groups during and after the PH-ST protocol (during 16–18 weeks of age, where PH-ST was administered from weeks 16–17 or days 29–35; β = *p* < 0.001.). However, the ANOVA-RM did not show an interaction effect of PH-ST, diet and time. Data are mean and standard error of the mean. SD, standard diet; HFD, high-fat diet. *** = *p* < 0.001.

Voluntary physical activity was decreased in the HFD group when compare to the SD group (Figure 6A) before the PH-ST protocol (Week 12–16). In fact, the ANOVA-RM revealed a main effect of diet (*F*_(__1_,_33__)_ = 72.67, *p* < 0.001), time (*F*_(__27_,_891__)_ = 46.26, *p* < 0.001), and an interaction effect of diet and time (*F*_(__27_,_891__)_ = 11.58, *p* < 0.001) on distance traveled daily. *Post hoc* analysis revealed from the 6th to 28th days that the HFD group had significantly decrease on distance traveled when compare to the SD group (Day 6: 1.32 ± 0.29 km vs. 4.57 ± 0.30 km, *p* = 0.001; Day 28: 2.51 ± 0.54 km vs. 8.17 ± 0.56 km, *p* = 0.0001, respectively). Further, to test whether PH-ST protocol provoked an effect on voluntary physical activity behavior, we performed an ANOVA-RM during and after PH-ST protocol. The analysis revealed a main effect of diet (*F*_(__1_,_31__)_ = 51.73, *p* < 0.001), time (*F*_(__13_,_403__)_ = 6.68, *p* < 0.001) and an interaction effect of diet and time (*F*_(__13_,_403__)_ = 2.80, *p* < 0.001) on distance traveled daily. However, the analysis did not reveal an interaction effect of diet and PH-ST (*F*_(__1_,_31__)_ = 2.49, *p* = 0.12), time and PH-ST (*F*_(__13_,_403__)_ = 1.09, *p* = 0.36) and diet, time and PH-ST (*F*_(__13_,_403__)_ = 0.28, *p* = 0.99) on distance traveled daily during and after the PH-ST protocol (Figure 6B).

### High-Fat Diet and Sedentary Behavior Promoted Chronic Pain Susceptibility

**FIGURE 7:**
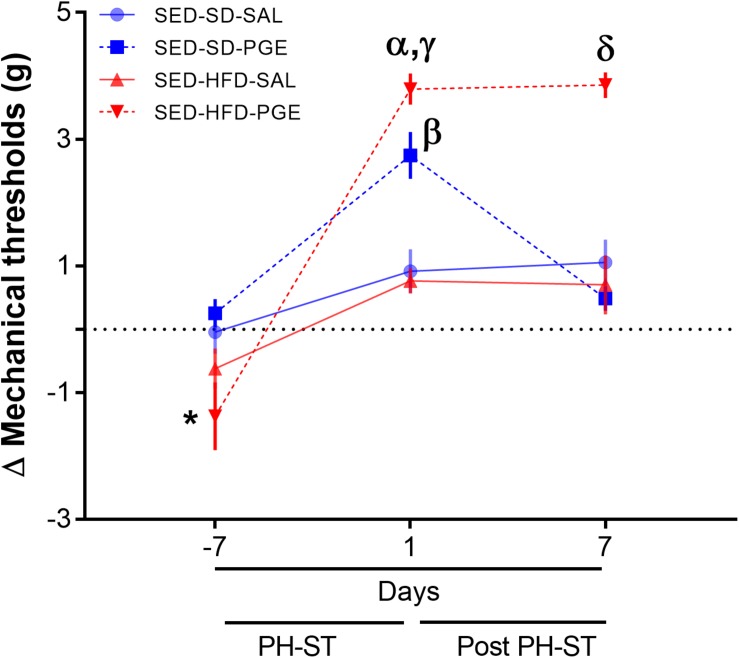
Chronic pain susceptibility promoted by HFD and sedentary behavior. The ANOVA-RM and *post hoc* analyses revealed on the 7th day after PH-ST protocol that the delta mechanical threshold was significantly higher in the SED-HFD-PGE group when compare to SED-SD-PGE, SED-HFD-SAL, and SED-SD-SAL groups. SED-SD-SAL and SED-HFD-SAL (*n* = 8), SED-SD-PGE and SED-HFD-PGE (*n* = 9). Data are mean and standard error of the mean. * = *p* < 0.05. SED-HFD-PGE ≠ SED-SD-PGE, SED-HFD-SAL, and SED-SD-SAL. α = *p* < 0.001. SED-HFD-PGE ≠ SED-SD-SAL and SED-HFD-SAL. γ = *p* = 0.02. SED-HFD-PGE ≠ SED-SD-PGE. β = *p* < 0.001 SED-SD-PGE ≠ SED-HFD-SAL and SED-SD-SAL. δ = *p* < 0.001 SED-HFD-PGE ≠ SED-SD-PGE, SED-HFD-SAL and SED-SD-SAL.

To test the effect of HFD and sedentary behavior on chronic pain susceptibility measured by the delta mechanical threshold, we first performed the ANOVA-RM test (diet vs. PH-ST vs. time) among groups (*N* = 4) assigned to sedentary behavior. The test revealed a main effect of PH-ST (*F*_(__1_,_30__)_ = 38.87, *p* < 0.001) and time (*F*_(__2_,_60__)_ = 61.08, *p* < 0.001), but no main effect of diet (*F*_(__1_,_30__)_ = 2.32, *p* = 0.137) on the delta mechanical threshold. On the other hand, the test revealed an interaction effect of diet and PH-ST (*F*_(__1_,_30__)_ = 11.94, *p* = 0.001) and of diet and time (*F*_(__2_,_60__)_ = 15.12, *p* < 0.001) on the delta mechanical threshold. Moreover, the ANOVA-RM also revealed an interaction effect of PH-ST, diet, and time (*F*_(__2_,_60__)_ = 12.52, *p* < 0.001) on the delta mechanical threshold (Figure 7). *Post hoc* analysis showed at 1 day before the beginning of PH-ST (−7 days Figure 7 or at week 16, vF Pre PH-ST, Figure 1) that the mean delta mechanical threshold of the SED-HFD-PGE group (−1.37 ± 0.53 g) was smaller when compared to SED-SD-PGE (0.25 ± 0.22 g, *p* = 0.004) and SED-SD-SAL (−0.04 ± 0.34 g, *p* = 0.016) groups, but not when compared to the SED-HFD-SAL (−0.82 ± 0.31 g, *p* = 0.113) group. Further, at the first day after PH-ST (1 days Figure 7 or at week 17, vF Post-PH-ST, Figure 1) *post hoc* analysis showed the mean delta mechanical threshold was significantly higher in the SED-HFD-PGE group (3.79 ± 0.24 g) when compared to SED-SD-PGE (2.74 ± 0.36, *p* = 0.028), SED-SD-SAL (0.91 ± 0.34, *p* = 0.0001), and SED-HFD-SAL (0.76 ± 0.19, *p* = 0.0001) groups. The mean delta mechanical threshold also was significantly higher in the SED-SD-PGE group when compare to SED-SD-SAL (*p* = 0.0006) and SED-HFD-SAL (*p* = 0.0004) groups. Finally, at the 7th day after PH-ST (7 days Figure 7 or at week 18, vF chronic pain susceptibility, Figure 1), *post hoc* analysis showed that the mean delta mechanical threshold was significantly higher only in the SED-HFD-PGE (3.85 ± 0.20) group when compare to SED-SD-PGE (0.48 ± 0.19 g, *p* = 0.0001), SED-HFD-SAL (0.70 ± 0.46 g, *p* = 0.0001), and SED-SD-SAL (1.05 ± 0.36 g, *p* = 0.0001) groups (Figure 7) suggesting an HFD combined with sedentary behavior promoted chronic pain susceptibility.

### Voluntary Physical Activity Prevented Chronic Pain Susceptibility

**FIGURE 8:**
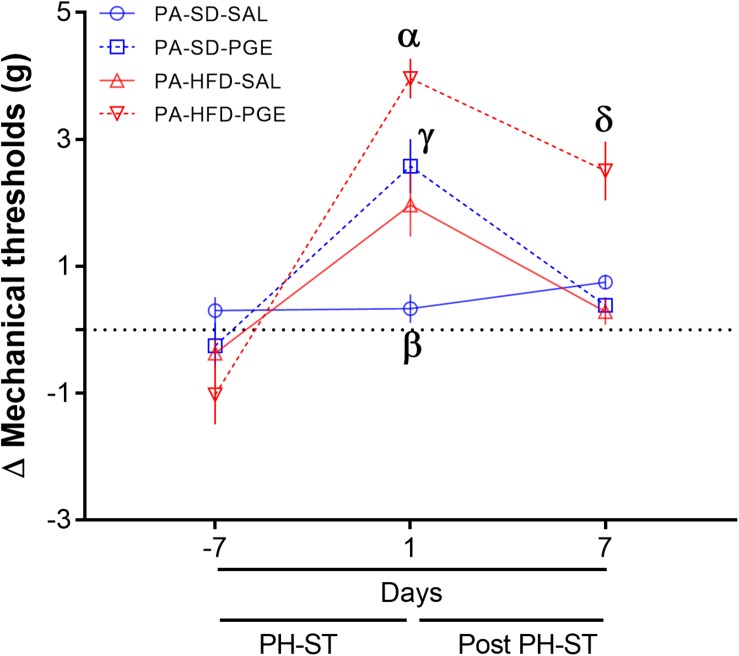
Physical activity prevented chronic pain susceptibility. The ANOVA-RM and *post hoc* analyses revealed on the 7th day after the PH-ST that the delta mechanical threshold was significantly decreased in the PA-HFD-PGE group when compared to the PA-HFD-PGE group at 1 day after PH-ST and was similar when compared to PA-SD-PGE and PA-HFD-SAL at 1 day after PH-ST. PA-HFD-PGE, PA-HFD-SAL, and PA-SD-PGE (*n* = 9). PA-SD-SAL (*n* = 8). Data are mean and standard error of the mean. α = *p* < 0.001. PA-HFD-PGE ≠ PA-SD-PGE, PA-HFD-SAL, and PA-SD-SAL. γ = *p* = 0.53. PA-HFD-SAL = PA-SD-PGE. β = *p* < 0.01. PA-SD-SAL ≠ PA-HFD-SAL. δ≠α = *p* = 0.008. PA-HFD-PGE. δ = γ = PA-HFD-PGE 7 days after PH-ST = PA-SD-PGE (*p* = 0.37) and PA-HFD-SAL (*p* = 0.84) 1 day after PH-ST. δ = *p* ≤ 0.001. PA-HFD-PGE ≠ PA-SD-PGE, PA-HFD-SAL and PA-SD-SAL.

To test the effect of physical activity on chronic pain susceptibility, we performed the same analyses described in section “High-Fat Diet and Sedentary Behavior Promoted Chronic Pain Susceptibility,” but here only among groups assigned to voluntary physical activity. The ANOVA-RM revealed a main effect of PH-ST (*F*_(__1_,_31__)_ = 19.47, *p* < 0.001), diet (*F*_(__1_,_31__)_ = 5.23, *p* = 0.02), and time (*F*_(__2_,_62__)_ = 60.28, *p* < 0.001) on the delta mechanical threshold. The test also revealed an interaction effect of time and diet (*F*_(__2_,_62__)_ = 16.19, *p* < 0.001), but no interaction effect of PH-ST and time (*F*_(__1_,_31__)_ = 1.90, *p* = 0.177) on the delta mechanical threshold. However, there was a significant interaction effect of PH-ST, time, and diet (*F*_(__2_,_62__)_ = 4.32, *p* = 0.017) on the delta mechanical threshold (Figure 8). In contrast to the effects reported above among the sedentary groups, the *post hoc* analysis showed at 1 day before the beginning of PH-ST (−7 days Figure 8 or at week 16, vF Pre PH-ST, Figure 1) that the mean delta mechanical threshold of the PA-HFD-PGE (−1.02 ± 0.46 g) group was not significantly different when to compare to all groups: PA-SD-PGE (−0.14 ± 0.33 g, *p* = 0.17), PA-HFD-SAL (−0.76 ± 0.43 g, *p* = 0.59), and PA-SD-SAL (0.32 ± 0.18 g, *p* = 0.056) groups. Further, at 1 day after PH-ST (1 day Figure 8 or week 17, vF Post-PH-ST, Figure 1), *post hoc* analysis showed the mean delta mechanical threshold was significantly higher in the PA-HFD-PGE group (3.95 ± 0.31 g) when compared to the PA-SD-PGE (2.6 ± 0.37 g, *p* = 0.007), PA-HFD-SAL (2.06 ± 0.44 g, *p* = 0.001), and PA-SD-SAL (0.15 ± 0.26 g, *p* = 0.0001) groups. Unexpectedly, the mean delta mechanical threshold was also significantly higher in the PA-HFD-SAL group when compared to the PA-SD-SAL group (*p* = 0.002) but was significantly smaller when compared to the PA-HFD-PGE group (*p* = 0.001). Finally, *post hoc* analysis showed that within the PA-HFD-PGE group, the mean delta mechanical threshold significantly decreased at 7 days after the PH-ST protocol versus the previous timepoint at day 1 after PH-ST (2.5 ± 0.46 g vs. 3.95 ± 31 g, *p* = 0.008). Moreover, 7 days after the PH-ST protocol – the chronic pain susceptibility measurement timepoint, the mean delta mechanical threshold of the PA-HFD-PGE group (2.5 ± 0.46 g) was similar to the 1 day after PH-ST-induced mean delta mechanical threshold of the PA-SD-PGE (2.6 ± 0.37 g, *p* = 0.84) and PA-HFD-SAL (2.06 ± 0.44 g, *p* = 0.37) groups (Figure 8). This suggests voluntary physical activity plays a role in preventing chronic pain susceptibility promoted by HFD and sedentary behavior. However, the analyses also showed that the mean delta mechanical threshold remained significantly higher at 7 days after the PH-ST protocol in the PA-HFD-PGE group when compared to the PA-SD-PGE (0.57 ± 0.21 g, *p* = 0.0006), PA-HFD-SAL (0.33 ± 0.19 g, *p* = 0.0004), and PA-SD-SAL (0.55 ± 0.21 g, *p* = 0.001) groups.

Further, to confirm the preventive effect of voluntary physical activity on chronic pain susceptibility, we performed the ANOVA-RM including both sedentary and physical activity groups. A significant interaction effect of PH-ST, time, physical activity behavior and diet was found (*F*_(__2_,_122__)_ = 3.14, *p* = 0.046) on the delta mechanical threshold including all eight groups. The *post hoc* analysis confirmed that the mean delta mechanical threshold was significantly lower in the PA-HFD-PGE, a non-chronic pain susceptibility group when compared to the SED-HFD-PGE, a chronic pain susceptibility group (2.5 ± 0.46 g Figure 8, vs. 2.06 ± 0.44 g Figure 7, *p* = 0.041) at 7 days after the PH-ST protocol – the chronic pain susceptibility measurement timepoint.

### Differential Gene Expression in the Nucleus Accumbens – Transcriptome Analysis

**FIGURE 9:**
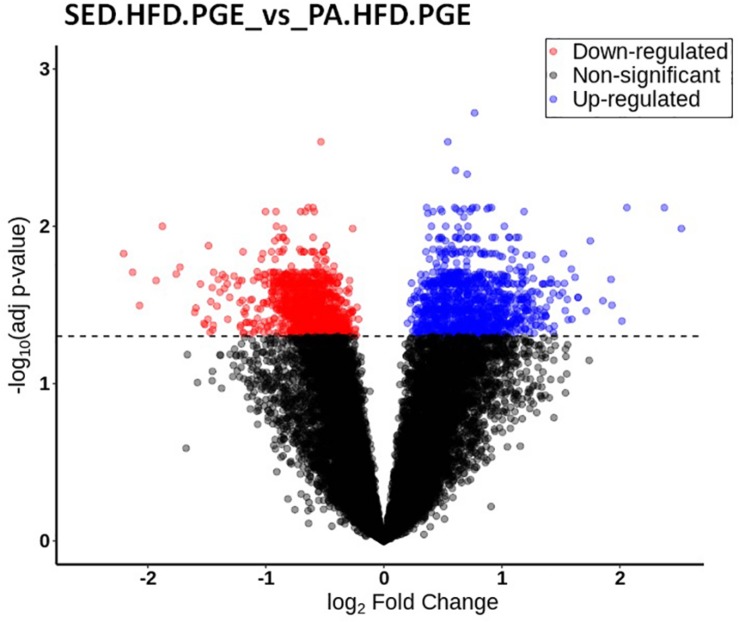
Volcano plot of the differential gene expression in the nucleus accumbens (NAc). The differential gene expression analysis of SED-HFD-PGE vs. PA-HFD-PGE groups revealed that 2,204 were differentially expressed in the NAc. Among those genes, 1,098 were up-regulated (blue) and 1,106 were down-regulated genes (in red) in the PA-HFD-PGE group when compared to SED-HFD-PGE group. Genes in black are not significantly different. Adjusted *p*-value *p* = 0.05.

To investigate the differential gene expression in the NAc related to chronic pain susceptibility promoted by an HFD and sedentary behavior and prevented by voluntary physical activity, we first compared the differential gene expression of the SED-HFD-PGE, a chronic pain susceptibility group (Figure 7) to the PA-HFD-PGE, a group that showed resistance to chronic pain susceptibility (Figure 8). The analysis revealed that 2,204 genes were differentially expressed in the NAc between the SED-HFD-PGE and PA-HFD-PGE group. Of the differentially expressed genes, 1,098 were significantly up-regulated and 1,106 down-regulated in the PA-HFD-PGE group compared to the SED-HFD-PGE group thereby identifying genes involved in preventing and promoting chronic pain susceptibility, respectively (Figure 9 and Supplementary Table S1).

**FIGURE 10:**
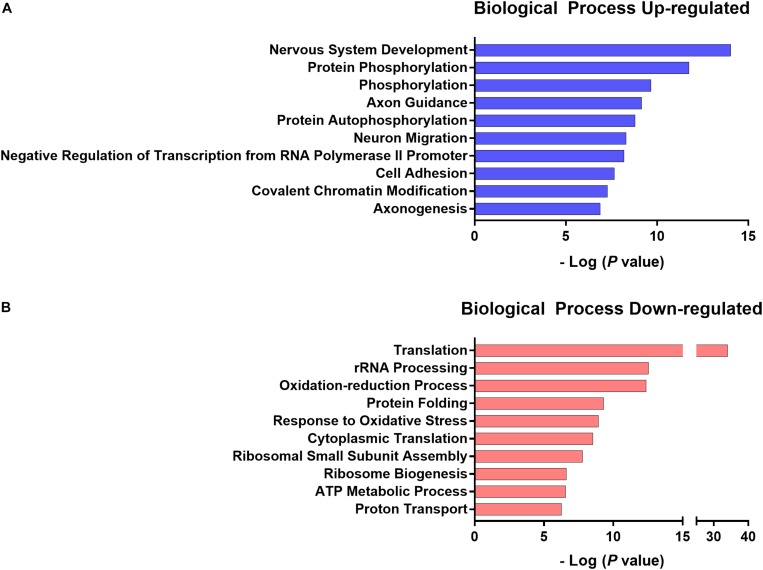
The 10 lowest *p*-value up- and down-regulated biological processes. The 10 lowest normalized *p*-value of biological processes up- **(A)** and down- **(B)** regulated genes related to chronic pain susceptibility induced by HFD and sedentary behavior and prevented by voluntary physical activity.

According to DAVID gene ontology enrichment results, these genes were implicated in 41 biological processes (EASE *p* < 0.001 and FDR *p* < 0.05), 23 biological processes were enriched when considering up-regulated genes and 18 were enriched when considering down-regulated genes. One biological process was both up- and down-regulated (GO:0006810 transport). The 10 lowest *p*-values from the up and down-regulated genes related to the biological processes are presented in Figure 10 and the gene list is presented in Tables 3, 4. The total list of biological processes and differential gene expression in the NAc implicated in chronic pain susceptibility promoted by an HFD and sedentary behavior in the SED-HFD-PGE group and prevented by voluntary physical activity (PA-HFD-PGE group) were described in the Supplementary Tables S2, S3.

**TABLE 3:** List of the biological processes and genes IDs of the six lowest *p*-value up-regulated genes enriched in the PA-HFD-PGE.

Biological processes	Genes ID	FEs	*p*-value	FDR *p*-value
Nervous System Development	NRP2, MTSS1, PLXNA4, GRIK1, GRIP1, FGF13, L1CAM, SLC7A5, APP, ROBO1, SMARCD1, DSCAM, NR2F1, TNIK, MAGI2, MDGA1, EFNB2, MBD5, ALK, MARK4, SLIT3, PRDM8, MYT1L, SLITRK1, DACT1, SEMA4G, NAV1, NAV2, EFNA5, SEMA4D, CNTN3, EIF2AK4, CAMK1D, KALRN, SMARCA4, SOX1, GLIS2, CAMK2G, BRSK2, DSCAML1, NEO1, EPHB2, ARX, CRMP1, NUMB, LMTK2, LHX6, D130043K22RIK, NKX2-2, PHACTR4, MAP1B, NTNG1, SHANK1, NTRK3, FNBP1, SEMA6D, EPHA8, SMARCC1, GSK3B, CIT, HDAC9	3.08	9.35E−15	1.71E−11
Protein Phosphorylation	CDK18, STK35, LATS1, D8ERTD82E, TGFB2, PAK6, ST3GAL1, ACVR1B, APP, HSF1, AAK1, WNK4, MAP3K9, CDK12, PRKACA, CDK16, INSR, CDK13, TNIK, STK24, WNK1, WNK2, ALK, PRKCE, MARK4, MARK1, MAST4, MAST1, KSR2, MAPK4, ERN1, PDGFRB, EIF2AK3, EIF2AK4, CAMK1D, KALRN, FGFR1, CDK5R1, ERBB3, CAMK2G, PEAK1, BRSK2, MKNK1, PXK, KIT, EPHB2, MAP3K3, MAP3K1, LMTK2, DCLK3, DYRK2, AATK, PDK2, SMAD9, FLT1, TGFBR2, TRIO, GAS6, FNIP2, KDR, NTRK3, PLK5, FYN, RPS6KA2, ULK1, EPHA8, GSK3B, CDC42BPA, MTOR, CIT, ABL1, GRK3, MYLK, CDC42BPB	2.45	1.79E−12	3.30E−9
Phosphorylation	CDK18, TOLLIP, STK35, PIP5K1C, VPRBP, LATS1, D8ERTD82E, PAK6, ACVR1B, WNK4, MAP3K9, CDK12, PRKACA, CDK16, INSR, CDK13, TNIK, STK24, WNK1, PI4KA, WNK2, ALK, PRKCE, MARK4, MARK1, MAST4, MAST1, KSR2, MAPK4, ERN1, PDGFRB, EIF2AK3, EIF2AK4, CAMK1D, KALRN, FGFR1, ADPGK, ERBB3, CAMK2G, PEAK1, BRSK2, MKNK1, KIT, EPHB2, MAP3K3, MAP3K1, LMTK2, DCLK3, CERK, DYRK2, AATK, PDK2, DGKQ, FLT1, TGFBR2, TRIO, SMG1, LRGUK, KDR, NTRK3, FYN, RPS6KA2, ULK1, EPHA8, GSK3B, CDC42BPA, MTOR, CIT, ABL1, GRK3, MYLK, CDC42BPB	2.24	2.18E−10	4.03E−7
Axon Guidance	ABLIM1, NRP2, CDK5R1, PLXNA4, ARHGAP35, L1CAM, NEO1, EPHB2, TGFB2, ARX, LAMB2, UNC5B, UNC5A, ANK3, CRMP1, ROBO1, LMTK2, EFNB2, NFASC, SLIT3, EPHA8, FYN, DLX5, TENM2, CNTN2, EFNA5, LAMC1, KIF26A, APBB2, MYH10	3.84	7.06E−10	1.30E−6
Protein Autophosphorylation	FGFR1, CAMK2G, PEAK1, MKNK1, KIT, ACVR1B, MAP3K3, MAP3K9, AAK1, CDK12, LMTK2, PRKACA, INSR, AATK, FLT1, TNIK, STK24, WNK1, SMG1, WNK2, ALK, KDR, NTRK3, EPHA8, ULK1, FYN, GSK3B, ERN1, PDGFRB, MTOR, ABL1, EIF2AK3, EIF2AK4	3.44	1.60E−9	2.94E−6
Neuron Migration	NRP2, CDK5R1, SOX1, FGF13, NEO1, CXCL12, ARX, FAT3, D130043K22RIK, NR2F2, NR2F1, PCNT, MDGA1, CELSR3, ITGA3, CELSR2, CELSR1, MARK1, GAS6, NTRK3, BBS1, NAV1, FYN, CNTN2, APBB2, MYH10	4.00	4.93E−9	1.99E−5

*FEs, fold enrichment score; FDR, false discovery rate.*

**TABLE 4:** List of the biological processes and genes IDs of the six lowest *p*-value up-regulated genes enriched in the SED-HFD-PGE group.

Biological processes	Genes ID	FEs	*p*-value	FDR *p*-value
Translation	RPL18, RPL17, RPL36A, RPL19, MRPL41, RPL14, RPL13, RPL15, RPS27L, RPL22L1, MRPL36, RPL10, FAU, RPL11, RPL12, RPS27A, 2810006K23RIK, RPL35A, MRPL52, MRPL51, RPS18, RPS19, SLC25A34, RPS16, RPS17, RPS14, RARS, RPS15, MRPL49, RPS12, GATB, MRPS17, EEF1B2, MRPS14, FARS2, MTIF3, QRSL1, RPS26, RPS27, RPS28, RPL7, RPS29, RPL6, RPL9, EIF3K, RPL3, MRPL55, EIF3L, EIF3I, RPL5, RPL10A, RPL7A, RPS20, RPS21, RPS23, EIF3M, RPS24, RPSA, MRPS24, RPL23A, MRPS21, DENR, RPS6, MRRF, RPS5, RPS8, RPS3A1, RPS7, SLC25A14, RPL18A, RPL37A, RPS3, MRPS5, RPS4X, RPL41, EIF4A1, FARSB, NHP2, RPL27A, RPL35, RPL36, RPS15A, RPL37, RPL38, RPL39, MRPL20, RACK1, MRPL11, RPL30, MRPL15, RPL32, RPL31, RPL34, MRPL18, RPS27RT, AIMP1, AIMP2, RPL26, RPL27, MRPL30, RPL29, MRPL24, MRPL23, MRPL21, RPL23, RPL22, RPL13A, RPL21	5.50	9.17E−50	1.62E−46
rRNA processing	RPL14, LSM6, FCF1, SBDS, IMP3, RPS28, RPL7, RPL5, RPL11, MTERF4, FTSJ3, RPS24, RPL35A, EXOSC9, EXOSC7, EMG1, RPL26, GTF2H5, RPS6, NOP10, RNMTL1, MRM2, RPS7, RPS19, PIH1D1, RPS16, RPS17, RPS15, POP5, MPHOSPH6, NHP2	5.02	2.96E−13	5.24E−10
Oxidation-Reduction Process	LDHB, LDHA, GMPR2, PRDX5, RPE65, PRDX2, UQCRQ, PRDX1, FDFT1, NDUFS6, GPX1, HMOX2, UQCR10, MSRA, NDUFS5, UQCR11, NDUFS4, IDH3G, HMOX1, GPX4, NDUFS8, NDUFS3, SUOX, NDUFB11, NDUFC2, CYB5A, DECR1, CBR3, COQ7, CDO1, DHRS7B, DDO, NDUFA12, NDUFA11, DHRS1, DHRS4, PRDX6, MARC2, UQCRH, UQCRB, PRODH, HSD17B11, NDUFB3, HSD17B10, NDUFB5, NDUFB6, NDUFB7, TXN2, NDUFB9, ADH5, TXN1, SESN1, HADHA, CYB561D2, AKR1A1, FMO1, FAM213A, NDUFA4, NDUFA5, NDUFA2, NDUFA3, NDUFA9, NDUFA6, NDUFA7, FAM213B, SOD1, NDUFA1, MSRB2, BLVRA, NDUFV3, SDHB, TXNDC12, AKR1B3, HSDL2, BLVRB, NDUFV2, PHGDH, CRYM, DCXR, MGST1	2.41	4.36E−13	7.72E−10
Protein Folding	FKBP8, GRPEL1, PDIA3, TXN2, FKBP4, PDIA6, TXN1, CCT3, HSCB, CDC37, PPIL3, PDRG1, HSPE1, TUBA1B, HSPA8, TCP1, DNLZ, PPIE, PFDN1, PPIH, HSP90B1, CCT5, PPIB, PPIA, PFDN5, TBCC, AHSA1	4.30	4.90E−10	8.68E−7
Response to Oxidative Stress	NDUFB4, ATOX1, PRDX5, PINK1, PRDX2, PRDX1, RPS3, PSMB5, GPX1, HMOX2, MSRA, APOE, GPX4, HMOX1, TOR1A, NDUFS8, ERCC1, MT3, SELK, NDUFA6, CST3, SOD1, COQ7, MSRB2, PARK7, NDUFA12, PEBP1	4.14	1.17E−9	2.07E−6
Cytoplasmic Translation	RPL35A, RPL15, RPL26, RPL36, RPL22L1, RPL29, RPL7, RPL31, RPL6, RPL22, RPL9, RPLP0, RPLP1, FTSJ1	8.41	2.97E−9	5.26E−6

*FEs, fold enrichment score; FDR, false discovery rate.*

Furthermore, the interaction factor analysis (diet, physical activity, and PH-ST) of the differential gene expression within the sedentary or within the voluntary physical activity groups (i.e., an intragroup analysis) did not reveal gene expression changes in the NAc. For instance, we compared the differential gene expression in the NAc of the SED-HFD-PGE, a chronic pain susceptibility group, versus the SED-SD-PGE, a non-chronic pain susceptibility group (Figure 7) and also compared the PA-HFD-PGE, a resistant chronic pain susceptibility group and versus the PA-SD-PGE, a non-chronic pain group (Figure 8) and, both analyses did not reveal any differential gene expression in the NAc. Confirming our hypothesis that chronic pain susceptibility was promoted when an HFD was combined with sedentary behavior and that voluntary physical activity *per se* prevented chronic pain susceptibility even when mice were fed an HFD.

## Discussion

First, we report that HFD and sedentary behavior promoted chronic pain susceptibility. Secondly, we also report that voluntary physical activity prevented chronic pain susceptibility even when mice were fed an HFD. Thirdly, the analysis of interaction factors of the differential gene expression in the NAc of the chronic pain susceptibility group (SED-HFD-PGE) and non-chronic pain susceptibility group (PA-HFD-PGE) suggested that these gene expression changes may be implicated in chronic pain susceptibility. Fourthly, the enrichment gene ontology analysis revealed that metabolic and mitochondrial stress biological processes were significantly enriched in the chronic pain susceptibility group (SED-HFD-PGE). Meanwhile, biological processes implicated in neuroplasticity were significantly enriched in the non-chronic pain susceptibility group (PA-HFD-PGE). Finally, these results confirmed our hypothesis that an HFD and sedentary behavior lead to chronic pain susceptibility, while voluntary physical activity can prevent it.

Our data showed that the PGE-induced PH-ST protocol induced chronic pain susceptibility in the SED-HFD-PGE group, but it was not enough to induce chronic pain susceptibility on sedentary mice fed an SD (SED-SD-PGE) and in the SED-HFD-SAL group (Figure 7). This result supports findings from Song et al. (2017) that HFD increases pain regardless of obesity status. Although Song et al. (2017) did not investigate an interaction effect of sedentary behavior on chronic pain susceptibility, they found an increased macrophage density in the dorsal root ganglia related to increase of pain behaviors and HFD intake. Moreover, previous studies have documented the role of HFD in nociception modulation and chronic pain in rodent models (Rossi et al., 2013; Cooper M. A. et al., 2018; Liang et al., 2019). For instance, Liang et al. (2019) demonstrated a microglial spinal cord reaction characterized by up-regulation of pro-inflammatory cytokines related to increase of nociceptive responses in HFD mice. In our study, although gene expression changes in the NAc microglial may not be inferred due the mixed cell population of the transcriptome approach applied, the results of mechanical nociceptive and gene expression changes corroborated these previous studies and added new insights on the effect of a HFD and sedentary behavior to promote in chronic pain susceptibility. Our data revealed new interaction factors of the gene expression changes in the NAc, a key structure in the chronic pain susceptibility (Dias et al., 2015; Schwartz et al., 2017; Zhang et al., 2019) associated with HFD and sedentary behavior.

Surprisingly, chronic pain susceptibility was prevented in the voluntary physical activity group (PA-HFD-PGE) (Figure 8), regardless of the low level of distance traveled when compared to standard diet groups (Figure 6). The data of the mean delta mechanical threshold from the PA-HFD-PGE group (Figure 8) was significantly lower when compared to the SED-HFD-PGE group (Figure 7) on the 7th day after the PH-ST protocol. Moreover, the mean delta mechanical threshold from the PA-HFD-PGE group was also significantly lower on the 7th day when compared to the first day after the PGE-induced PH-ST protocol (Figure 8), suggesting, thus, the effect of voluntary physical activity in chronic pain susceptibility prevention. Although the mean delta mechanical threshold of the PA-HFD-PGE group has remained significantly increased when compared to PA-SD-PGE, PA-HFD-SAL, and PA-SD-SAL groups at the 7th day after the end of PGE-induced PH-ST protocol, this data showed that voluntary physical activity was enough to prevent chronic pain susceptibility promoted by HFD and sedentary behavior. Further studies including a new mechanical threshold test 14 days after the end of PGE-induced PH-ST protocol on both, sedentary and voluntary physical activity cohorts, could be interesting and reveal long-lasting effect of the voluntary physical activity on chronic pain prevention.

Another interesting question raised from our results that could lead to new findings, but presents methodologically challenges, would be to “motivate” the HFD-fed mice to do, voluntarily, the same activity level than SD-fed mice. In fact, Friend et al. (2017) showed that HFD contributes to physical inactivity in mice with obesity and it was independent of body mass or obesity *per se*, but associated to striatal dysfunction of dopamine signaling; namely the mice showed a diet-induced lack of motivation for voluntary physical activity. Other researchers have already shown a decrease of motivation and distance traveled in voluntary physical activity when mice were fed an HFD or a typical western diet compared to mice fed a standard diet (Funkat et al., 2004; Bjursell et al., 2008; Vellers et al., 2017). However, the differential gene expression in the NAc related to motivation for physical inactivity is unknown and few studies investigated these questions looking the NAc, a key structure known for motivation (Salamone and Correa, 2012; Salamone et al., 2015).

The analysis of interaction factors from the transcriptome of the NAc revealed gene expression changes were implicated in preventing and promoting chronic pain susceptibility (Figure 9). Although our study has been the first to employ a transcriptomic approach of the NAc to investigate chronic pain promoted by an HFD and sedentary behavior, other studies have shown that differential gene expression in the NAc was implicated in chronic pain and other comorbidities. For instance, applying a transcriptomic approach to the NAc, mPFC, and PAG in mice, Descalzi et al. (2017) showed that neuropathic pain promoted gene expression changes in these brain areas and these changes were involved in stress and depression. Curiously, some biological processes implicated in neuropathic pain, stress and depression described by Descalzi et al. (2017) were similarly implicated in chronic pain susceptibility in our study, such as phosphorylation (GO:0016310) and regulation of transcription from RNA polymerase II promoter (GO:0000122) (Figure 10A). Thus, although Descalzi et al. (2017) did not investigate the effect of an HFD, sedentary behavior and voluntary physical activity in stress and depression, we reported here that these variables play a crucial role in chronic pain susceptibility and may also play a crucial role in stress and depression, but this question still needs further investigation.

In our study, the gene ontology enrichment analysis of differential gene expression in the NAc identified 41 biological processes that maybe implicated in chronic pain susceptibility (Figure 10B and Supplementary Tables S2, S3). Among these, 23 biological processes were significantly up-regulated in the SED-HFD-PGE group, a sedentary HFD and chronic pain susceptibility group (Table 4 and Supplementary Table S3). From these 23 biological processes significantly enriched, we found translation (GO:0006412), response to oxidative stress (GO:0006979), ATP metabolic process (GO:0046034), response to oxidative stress (GO:0006979), proton transport (GO:0015992) and, ATP synthesis couple proton transport (GO:0015986) processes, suggesting that genes implicated in metabolic and mitochondrial stress in the NAc may also be implicated in chronic pain susceptibility promotion. Moreover, the gene ontology enrichment analysis also identified 18 biological processes significantly up-regulated in the PA-HFD-PGE group, a voluntary physical activity HFD and non-chronic pain susceptibility group (Figure 10A, Table 3 and Supplementary Table S2). Among these 18 biological processes significantly enriched, we found nervous system development (GO:0007399), neuron migration (GO:0001764), axonogenesis (GO:0007409), protein autophosphorylation (GO:0006468), cell adhesion (GO:0007155), actin cytoskeleton organization (GO:0030036), and learning (GO:0007612) processes, suggesting that genes implicated in neuroplasticity in the NAc may also be implicated in chronic pain susceptibility prevention, at least in the HFD context.

Indeed, we were the first to report that biological processes involved in neuroplasticity, within an HFD and sedentary behavior context, may prevent chronic pain susceptibility. In fact, several studies reported that genes implicated in the neuroplasticity in the NAc is related to chronic pain facilitation and hyperalgesia in humans and animals (Woolf, 2000; Apkarian et al., 2011; Mansour et al., 2014; Zhang et al., 2014; Kuner and Flor, 2016; DosSantos et al., 2017). However, it is important to emphasize that studies with humans from functional magnetic resonance imaging showed evidence that chronic pain and neuroplasticity are a cognitive maladaptive learning process (Apkarian et al., 2011; Mansour et al., 2014; DosSantos et al., 2017). Whereas studies with animal models investigated the role of one single molecular signaling related to neuroplasticity, such as BDNF/TrkB pathway, for instance (Zhang et al., 2014, 2017; Wang et al., 2017). This controversy associating neuroplasticity to chronic pain facilitation or chronic pain susceptibility prevention, may be explained, in our study, by the early gene expression changes in the NAc caused by HFD employed 6 weeks before the voluntary physical activity period. First, likely, the genes implicated in metabolic and mitochondrial stress biologic processes, as described here, might have already unregulated functional activity within the NAc and, consequently, promoted chronic pain susceptibility. Further, as the voluntary physical activity can provoke multiples neuroplastic molecular signaling changes in the brain, the neuroplasticity found here might be the result of a more complex and coordinated neurobiological signaling than one single molecular signaling, such as the BDNF/TrkB pathway. On the other hand, voluntary physical activity and exercise are already known to produce neuroplasticity throughout several brain areas and through multiples neuroplastic molecular signaling pathways (Chen et al., 2017; Fernandes et al., 2017; Friend et al., 2017; Lourenco et al., 2019). Thereby, to the best of our knowledge, our study was the first to report that biological processes in the NAc implicated in metabolic and mitochondrial stress, and neuroplasticity, may also be implicated in promotion and prevention of chronic pain susceptibility, respectively.

Although our study did not indicate decreased body mass in the voluntary physical activity groups (Figure 2), a meta-analysis reported that body mass reduction promoted by physical activity or other interventions such as caloric restriction can contribute to pain reduction in humans with chronic musculoskeletal pain (Cooper L. et al., 2018). The contrast between these results may suggests that the effect of voluntary physical activity in preventing chronic pain susceptibility in mice, or pain reduction in humans with chronic pain, may also be mediated by differential gene expression related to neuroplasticity in the NAc, independent of body mass reduction, as was reported here. Despite Sluka et al. (2013) and Leung et al. (2016) have described the effects of regular physical activity to prevent chronic pain development in animals models, our study was the first to report the interaction between diet (SD or HFD), physical activity status (active or sedentary), and chronic pain susceptibility. Thus, taken together, our results may offer novel insights into molecular mechanisms and biological processes underlying the chronic pain susceptibility among individuals with obesity and sedentary lifestyles.

As expected and widely reported in the literature (Montgomery et al., 2013; Williams et al., 2014; Yang et al., 2014; Kleinert et al., 2018), after 12 weeks on an HFD, the body mass was significantly higher in the HFD group compared to the SD group (Figure 2). However, our results showed that 6 weeks of voluntary physical activity was not enough to reduce body mass in the physically active groups when compare to their sedentary counterparts, independent of the diet (HFD or SD) (Figures 2, 3). The body mass result reported here is in accordance with Rocha-Rodrigues et al. (2018), once they revealed no significant difference in rats body mass submitted in 8 weeks of running protocol (treadmill exercise or voluntary physical activity) after 9 weeks on an HFD. It suggests that whether mice start voluntary physical activity belatedly (i.e., 6 weeks in our study after initiating an HFD), the voluntary physical activity tends to produce no effect on the total body mass or in the amount of adipose tissue. As the aim of this study was to investigate the effect of an HFD, sedentary behavior and voluntary physical activity in the promotion or prevention of chronic pain susceptibility, the body mass stability throughout the study (Figures 3A,B), even after the physical activity period, increased our confidence that chronic pain susceptibility results are not related to body mass changes. Change in chronic pain status regardless of weight loss in animals fed an HFD was also reported by Song et al. (2017). This may suggest that even low levels of voluntary physical activity, despite no change in body mass, could be helpful and highly recommended as a complementary treatment for individuals with chronic pain and/or with obesity, due the potential gene expression changes in the NAc and other brain areas.

The caloric intake was higher in the HFD versus the SD group (Figure 4) and this result is in accordance with the literature (Yang et al., 2014; Hu S. et al., 2018; Kleinert et al., 2018). The high energy density found in the HFD (5.439 kcal/g) compared to the SD (3.080 kcal/g), particularly from fat (58.2% vs. 11.7%, respectively), support the increased caloric intake in the HFD group. This result is in accordance to a recent study where the authors suggested that the amount of fat from an HFD, but not protein or carbohydrate content, is the cause of increased adiposity in mice (Hu S. et al., 2018). Further, we also found distinct body composition, with increased adipose tissue in the HFD groups when compared to SD groups from retroperitoneal (Figure 5A) and epididymal adipose tissue (Figure 5B). Nonetheless, an unexpected interaction effect of voluntary physical activity and HFD in the epididymal tissue was found. We found a significantly higher amount of epididymal adipose tissue in the PA-HFD group when compared to the SD-HFD group (Figure 5B). Unlike our findings, a previous study showed that forced physical exercise on a treadmill was associated with epididymal adipose tissue decrease when mice were fed an SD (Castellani et al., 2014). However, conversely, Rocha-Rodrigues et al. (2018) reported no difference in epididymal adipose tissue when mice were fed an HFD 9 weeks before the start of a voluntary physical activity protocol.

The changes on the inflammatory profile of adipose tissue may also play a role in chronic pain susceptibility and acute nociception at a peripherical level. For instance, Hu Z. J. et al. (2018) demonstrated that increases on serum leptin in mice with obesity, an adipocytokine produced by white adipose tissue, decreased nociceptive response (i.e., cumulative flinches number) in both phases of a formalin-induced nociception model. In addition, Li et al. (2013) demonstrated that intrathecal leptin administration alleviated neuropathic pain induced by sciatic chronic constriction. Thus, our results raised other interesting and unanswered question on whether gene expression changes found in the NAc are modulated by inflammatory cytokines changes at the peripherical level.

## Conclusion

To conclude, first, we reported here that an HFD and sedentary behavior promoted chronic pain susceptibility in mice, whereas voluntary physical activity prevented it. Secondly, differential gene expression and gene ontology enrichment in the NAc analyses revealed that biological processes implicated in metabolic and mitochondrial stress were involved in promoting chronic pain susceptibility. We also concluded that biological processes implicated in neuroplasticity in the NAc supported chronic pain prevention. Thus, these findings suggested that even low levels of voluntary physical activity would be helpful and highly recommended as a complementary treatment for those individuals with chronic pain due to potential gene expression changes in the NAc, regardless of individual obesity status.

## Limitations

One of the limitations of our study was the lack of female mice groups to investigate potential sex differences on the effects of HFD and sedentary behavior in promoting chronic pain susceptibility, as well as the effect of voluntary physical activity to prevent it. Moreover, the lack of additional molecular approaches, such as knockout or knockdown, to test and/or confirm the role of some target genes does not allow the establishment of causality and the direct effects of variables investigated here. In addition, it would be interesting to consider other pain sensory measurement, such as thermal nociception or further measurement of mechanical nociception, however, to avoid bias a separated cohort of animals would be needed. Despite those limitations, we support that the strict methodological and statistical approaches employed here, as well as the findings reported, fulfill crucial scientific questions and provide new avenues for further studies.

## Data Availability Statement

The datasets generated for this study can be found here: http://www.ncbi.nlm.nih.gov/bioproject/564336.

## Ethics Statement

The animal study was reviewed and approved by the Animal Ethics Committee at the University of Campinas under protocol number 4243-1.

## Author Contributions

AB, CS, AV, and CP: conception of the project. AB, CS, and AV: design of the study and analysis and interpretations of the data. AB, IB, MP, and GZ: acquisition of the data. NP and AV: statistical and script programming support for RNA-Seq data analysis. AB and CS: drafting the manuscript. AB, IB, MP, GZ, CT, CP, NP, AV, and CS: final version approved. CS, AV, CT, and CP developed research with laboratory resources and equipment.

## Conflict of Interest

The authors declare that the research was conducted in the absence of any commercial or financial relationships that could be construed as a potential conflict of interest.

## Footnotes

1 ensembl.org

2 https://david.ncifcrf.gov/

## Supplementary Material

TABLE S1 Differentially expressed genes. SED-HFD-PGE_vs_PA-HFD-PGE.

TABLE S2 Up-regulated biological processes. PA-HFD-PGE.

TABLE S3 Up-regulated biological processes. SED-HFD-PGE.

DATA SHEET S1 DESeq2_R-script.
