# Supplementary material for: Physical Activity Induces Nucleus Accumbens Genes Expression Changes Preventing Chronic Pain Susceptibility Promoted by High-Fat Diet and Sedentary Behavior in Mice
Source: Front Neurosci. 2020 Jan 22;13:1453. doi: 10.3389/fnins.2019.01453 (PMC6987254; doi:10.3389/fnins.2019.01453)
Supplement: DATA SHEET S1 — DESeq2_R-script. [file Data_Sheet_1.PDF]

```
#References:
# 1) Love MI, Anders S, Kim V, Huber W (2016). RNA-Seq workflow: gene-level exploratory analysis and
differential
# expression. F1000Research 4:1070 Available at: https://f1000research.com/articles/4-1070/v2.

# 2) Love MI, Huber W, Anders S (2014). Moderated estimation of fold change and dispersion for RNA-seq data
with
# DESeq2. Genome Biol 15:1–21

#(https://www.bioconductor.org/packages/devel/bioc/vignettes/EnhancedVolcano/inst/doc/EnhancedVolcano.html)

################ Start ##############

library(DESeq2)
library(ggplot2)
library(pheatmap)
library(RColorBrewer)
library(genefilter)
library(EnhancedVolcano)
library(gridExtra)
library(gridBase)
library(STRINGdb)
library(tidyverse)
library(ggrepel)

file <- read.csv("/home/afb/AL3_data/AL3_data.csv", sep = ",", row.names = "gene")
head (file)

AL3_matrix <- as.matrix(file)#cria uma matrix para ser lida pelo DESeq
head(AL3_matrix)
coldata_AL3 <- read.csv("/home/afb/AL3_data/col_data_AL3.csv", sep = ",", row.names = "samples")

########
coldata_AL3 <- coldata_AL3[,c("treat", "rw", "diet")]
########

head (coldata_AL3)
summary (coldata_AL3)
all(rownames(coldata_AL3) %in% colnames(AL3_matrix))
all(rownames(coldata_AL3) == colnames(AL3_matrix))

############################
# 1.3.3 Count matrix input

dds <- DESeqDataSetFromMatrix(countData = AL3_matrix, colData = coldata_AL3, design = ~ treat + rw + diet)
dds

# 1.3.6 Pre-filtering and rlog normalization
dds <- dds[ rowSums(counts(dds)) > 0.9, ]
dds
colData(dds)
vsd_dds <- vst(dds)
##############################################


######### DESeq2 ######################################
#1.4 Differential expression analysis
dds <- DESeq(dds)
########################################

#----------------------------------#------------------------------------------#------------------------------------------#
colData(dds)
```

```
ddsIF_3 <- dds
ddsIF_3$group <- factor(paste0(ddsIF_3$rw, ddsIF_3$diet, ddsIF_3$treat))
design(ddsIF_3) <- ~ group
ddsIF_3 <- DESeq(ddsIF_3)
resultsNames(ddsIF_3)

#---- write table---#
write.csv(as.data.frame(raw_ddsIF_3), file = "/home/afb/AL3_data/raw_ddsIF_3.csv")
write.csv(as.data.frame(means_ddsIF_3), file = "/home/afb/AL3_data/means_ddsIF_3.csv")
write.csv(as.data.frame(cooks_ddsIF_3), file = "/home/afb/AL3_data/cooks_ddsIF_3.csv")
#---------------------#

#### 1)  SED.SD.SAL vs PA.HFD.SAL ######
SED.SD.SAL_PA.HFD.SAL <- results(ddsIF_3, contrast = c("group", "SEDSDSAL", "PAHFDSAL" ))
SED.SD.SAL_PA.HFD.SAL <- SED.SD.SAL_PA.HFD.SAL[order(SED.SD.SAL_PA.HFD.SAL$padj),]
SED.SD.SAL_PA.HFD.SAL
summary(SED.SD.SAL_PA.HFD.SAL)
sum(SED.SD.SAL_PA.HFD.SAL$padj < 0.1, na.rm = TRUE)
sum(SED.SD.SAL_PA.HFD.SAL$padj < 0.05, na.rm = TRUE)
write.csv(as.data.frame(SED.SD.SAL_PA.HFD.SAL), file =
"/home/afb/AL3_data/AL3_SED.SD.SAL_PA.HFD.SAL.csv")

#### 2)  SED.SD.SAL vs PA.HFD.PGE ######
SED.SD.SAL_PA.HFD.PGE <- results(ddsIF_3, contrast = c("group", "SEDSDSAL", "PAHFDPGE" ))
SED.SD.SAL_PA.HFD.PGE <- SED.SD.SAL_PA.HFD.PGE[order(SED.SD.SAL_PA.HFD.PGE$padj),]
SED.SD.SAL_PA.HFD.PGE
summary(SED.SD.SAL_PA.HFD.PGE)
sum(SED.SD.SAL_PA.HFD.PGE$padj < 0.1, na.rm = TRUE)
sum(SED.SD.SAL_PA.HFD.PGE$padj < 0.05, na.rm = TRUE)
write.csv(as.data.frame(SED.SD.SAL_PA.HFD.PGE), file =
"/home/afb/AL3_data/AL3_SED.SD.SAL_PA.HFD.PGE.csv")

#### 3)  SED.SD.PGE vs PA.HFD.SAL ######
SED.SD.PGE_PA.HFD.SAL <- results(ddsIF_3, contrast = c("group", "SEDSDPGE", "PAHFDSAL" ))
SED.SD.PGE_PA.HFD.SAL <- SED.SD.PGE_PA.HFD.SAL[order(SED.SD.PGE_PA.HFD.SAL$padj),]
SED.SD.PGE_PA.HFD.SAL
summary(SED.SD.PGE_PA.HFD.SAL)
sum(SED.SD.PGE_PA.HFD.SAL$padj < 0.1, na.rm = TRUE)
sum(SED.SD.PGE_PA.HFD.SAL$padj < 0.05, na.rm = TRUE)
write.csv(as.data.frame(SED.SD.PGE_PA.HFD.SAL), file =
"/home/afb/AL3_data/AL3_SED.SD.PGE_PA.HFD.SAL.csv")

#### 4)  SED.SD.PGE vs PA.HFD.PGE ######
SED.SD.PGE_PA.HFD.PGE <- results(ddsIF_3, contrast = c("group", "SEDSDPGE", "PAHFDPGE" ))
SED.SD.PGE_PA.HFD.PGE <- SED.SD.PGE_PA.HFD.PGE[order(SED.SD.PGE_PA.HFD.PGE$padj),]
SED.SD.PGE_PA.HFD.PGE
summary(SED.SD.PGE_PA.HFD.PGE)
sum(SED.SD.PGE_PA.HFD.PGE$padj < 0.1, na.rm = TRUE)
sum(SED.SD.PGE_PA.HFD.PGE$padj < 0.05, na.rm = TRUE)
write.csv(as.data.frame(SED.SD.PGE_PA.HFD.PGE), file =
"/home/afb/AL3_data/AL3_SED.SD.PGE_PA.HFD.PGE.csv")

#### 5)  SED.SD.SAL vs PA.SD.SAL ######
SED.SD.SAL_PA.SD.SAL <- results(ddsIF_3, contrast = c("group", "SEDSDSAL", "PASDSAL" ))
SED.SD.SAL_PA.SD.SAL <- SED.SD.SAL_PA.SD.SAL[order(SED.SD.SAL_PA.SD.SAL$padj),]
SED.SD.SAL_PA.SD.SAL
summary(SED.SD.SAL_PA.SD.SAL)
sum(SED.SD.SAL_PA.SD.SAL$padj < 0.1, na.rm = TRUE)
sum(SED.SD.SAL_PA.SD.SAL$padj < 0.05, na.rm = TRUE)
write.csv(as.data.frame(SED.SD.SAL_PA.SD.SAL), file =
"/home/afb/AL3_data/AL3_SED.SD.SAL_PA.SD.SAL.csv")
```

```
#### 6)  SED.SD.SAL vs PA.SD.PGE ######
SED.SD.SAL_PA.SD.PGE <- results(ddsIF_3, contrast = c("group", "SEDSDSAL", "PASDPGE" ))
SED.SD.SAL_PA.SD.PGE <- SED.SD.SAL_PA.SD.PGE[order(SED.SD.SAL_PA.SD.PGE$padj),]
SED.SD.SAL_PA.SD.PGE
summary(SED.SD.SAL_PA.SD.PGE)
sum(SED.SD.SAL_PA.SD.PGE$padj < 0.1, na.rm = TRUE)
sum(SED.SD.SAL_PA.SD.PGE$padj < 0.05, na.rm = TRUE)
write.csv(as.data.frame(SED.SD.SAL_PA.SD.PGE), file =
"/home/afb/AL3_data/AL3_SED.SD.SAL_PA.SD.PGE.csv")

#### 7)  SED.SD.PGE vs PA.SD.SAL ######
SED.SD.PGE_PA.SD.SAL <- results(ddsIF_3, contrast = c("group", "SEDSDPGE", "PASDSAL" ))
SED.SD.PGE_PA.SD.SAL <- SED.SD.PGE_PA.SD.SAL[order(SED.SD.PGE_PA.SD.SAL$padj),]
SED.SD.PGE_PA.SD.SAL
summary(SED.SD.PGE_PA.SD.SAL)
sum(SED.SD.PGE_PA.SD.SAL$padj < 0.1, na.rm = TRUE)
sum(SED.SD.PGE_PA.SD.SAL$padj < 0.05, na.rm = TRUE)
write.csv(as.data.frame(SED.SD.PGE_PA.SD.SAL), file =
"/home/afb/AL3_data/AL3_SED.SD.PGE_PA.SD.SAL.csv")

#### 8)  SED.SD.PGE vs PA.SD.PGE ######
SED.SD.PGE_PA.SD.PGE <- results(ddsIF_3, contrast = c("group", "SEDSDPGE", "PASDPGE" ))
SED.SD.PGE_PA.SD.PGE <- SED.SD.PGE_PA.SD.PGE[order(SED.SD.PGE_PA.SD.PGE$padj),]
SED.SD.PGE_PA.SD.PGE
summary(SED.SD.PGE_PA.SD.PGE)
sum(SED.SD.PGE_PA.SD.PGE$padj < 0.1, na.rm = TRUE)
sum(SED.SD.PGE_PA.SD.PGE$padj < 0.05, na.rm = TRUE)
write.csv(as.data.frame(SED.SD.PGE_PA.SD.PGE), file =
"/home/afb/AL3_data/AL3_SED.SD.PGE_PA.SD.PGE.csv")

#### 9)  SED.HFD.SAL vs PA.SD.SAL ######
SED.HFD.SAL_PA.SD.SAL <- results(ddsIF_3, contrast = c("group", "SEDHFDSAL", "PASDSAL" ))
SED.HFD.SAL_PA.SD.SAL <- SED.HFD.SAL_PA.SD.SAL[order(SED.HFD.SAL_PA.SD.SAL$padj),]
SED.HFD.SAL_PA.SD.SAL
summary(SED.HFD.SAL_PA.SD.SAL)
sum(SED.HFD.SAL_PA.SD.SAL$padj < 0.1, na.rm = TRUE)
sum(SED.HFD.SAL_PA.SD.SAL$padj < 0.05, na.rm = TRUE)
write.csv(as.data.frame(SED.HFD.SAL_PA.SD.SAL), file =
"/home/afb/AL3_data/AL3_SED.HFD.SAL_PA.SD.SAL.csv")

#### 10)  SED.HFD.SAL vs PA.SD.PGE ######
SED.HFD.SAL_PA.SD.PGE <- results(ddsIF_3, contrast = c("group", "SEDHFDSAL", "PASDPGE" ))
SED.HFD.SAL_PA.SD.PGE <- SED.HFD.SAL_PA.SD.PGE[order(SED.HFD.SAL_PA.SD.PGE$padj),]
SED.HFD.SAL_PA.SD.PGE
summary(SED.HFD.SAL_PA.SD.PGE)
sum(SED.HFD.SAL_PA.SD.PGE$padj < 0.1, na.rm = TRUE)
sum(SED.HFD.SAL_PA.SD.PGE$padj < 0.05, na.rm = TRUE)
write.csv(as.data.frame(SED.HFD.SAL_PA.SD.PGE), file =
"/home/afb/AL3_data/AL3_SED.HFD.SAL_PA.SD.PGE.csv")

#### 11)  SED.HFD.PGE vs PA.SD.SAL ######
SED.HFD.PGE_PA.SD.SAL <- results(ddsIF_3, contrast = c("group", "SEDHFDPGE", "PASDSAL" ))
SED.HFD.PGE_PA.SD.SAL <- SED.HFD.PGE_PA.SD.SAL[order(SED.HFD.PGE_PA.SD.SAL$padj),]
SED.HFD.PGE_PA.SD.SAL
summary(SED.HFD.PGE_PA.SD.SAL)
sum(SED.HFD.PGE_PA.SD.SAL$padj < 0.1, na.rm = TRUE)
sum(SED.HFD.PGE_PA.SD.SAL$padj < 0.05, na.rm = TRUE)
write.csv(as.data.frame(SED.HFD.PGE_PA.SD.SAL), file =
"/home/afb/AL3_data/AL3_SED.HFD.PGE_PA.SD.SAL.csv")

#### 12)  SED.HFD.PGE vs PA.SD.PGE ######
SED.HFD.PGE_PA.SD.PGE <- results(ddsIF_3, contrast = c("group", "SEDHFDPGE", "PASDPGE" ))
```

```
SED.HFD.PGE_PA.SD.PGE <- SED.HFD.PGE_PA.SD.PGE[order(SED.HFD.PGE_PA.SD.PGE$padj),]
SED.HFD.PGE_PA.SD.PGE
summary(SED.HFD.PGE_PA.SD.PGE)
sum(SED.HFD.PGE_PA.SD.PGE$padj < 0.1, na.rm = TRUE)
sum(SED.HFD.PGE_PA.SD.PGE$padj < 0.05, na.rm = TRUE)
write.csv(as.data.frame(SED.HFD.PGE_PA.SD.PGE), file =
"/home/afb/AL3_data/AL3_SED.HFD.PGE_PA.SD.PGE.csv")

#### 13)  SED.SD.SAL vs SED.SD.PGE ######
SED.SD.SAL_SED.SD.PGE <- results(ddsIF_3, contrast = c("group", "SEDSDSAL", "SEDSDPGE" ))
SED.SD.SAL_SED.SD.PGE <- SED.SD.SAL_SED.SD.PGE[order(SED.SD.SAL_SED.SD.PGE$padj),]
SED.SD.SAL_SED.SD.PGE
summary(SED.SD.SAL_SED.SD.PGE)
sum(SED.SD.SAL_SED.SD.PGE$padj < 0.1, na.rm = TRUE)
sum(SED.SD.SAL_SED.SD.PGE$padj < 0.05, na.rm = TRUE)
write.csv(as.data.frame(SED.SD.SAL_SED.SD.PGE), file =
"/home/afb/AL3_data/AL3_SED.SD.SAL_SED.SD.PGE.csv")

#### 14)  SED.HFD.SAL vs SED.HFD.PGE ######
SED.HFD.SAL_SED.HFD.PGE <- results(ddsIF_3, contrast = c("group", "SEDHFDSAL", "SEDHFDPGE" ))
SED.HFD.SAL_SED.HFD.PGE <-
SED.HFD.SAL_SED.HFD.PGE[order(SED.HFD.SAL_SED.HFD.PGE$padj),]
SED.HFD.SAL_SED.HFD.PGE
summary(SED.HFD.SAL_SED.HFD.PGE)
sum(SED.HFD.SAL_SED.HFD.PGE$padj < 0.1, na.rm = TRUE)
sum(SED.HFD.SAL_SED.HFD.PGE$padj < 0.05, na.rm = TRUE)
write.csv(as.data.frame(SED.HFD.SAL_SED.HFD.PGE), file =
"/home/afb/AL3_data/AL3_SED.HFD.SAL_SED.HFD.PGE.csv")

#### 15)  SED.SD.SAL vs SED.HFD.SAL ######
SED.SD.SAL_SED.HFD.SAL <- results(ddsIF_3, contrast = c("group", "SEDSDSAL", "SEDHFDSAL" ))
SED.SD.SAL_SED.HFD.SAL <- SED.SD.SAL_SED.HFD.SAL[order(SED.SD.SAL_SED.HFD.SAL$padj),]
SED.SD.SAL_SED.HFD.SAL
summary(SED.SD.SAL_SED.HFD.SAL)
sum(SED.SD.SAL_SED.HFD.SAL$padj < 0.1, na.rm = TRUE)
sum(SED.SD.SAL_SED.HFD.SAL$padj < 0.05, na.rm = TRUE)
write.csv(as.data.frame(SED.SD.SAL_SED.HFD.SAL), file =
"/home/afb/AL3_data/AL3_SED.SD.SAL_SED.HFD.SAL.csv")

#### 16)  SED.SD.PGE vs SED.HFD.PGE ######
SED.SD.PGE_SED.HFD.PGE <- results(ddsIF_3, contrast = c("group", "SEDSDPGE", "SEDHFDPGE" ))
SED.SD.PGE_SED.HFD.PGE <- SED.SD.PGE_SED.HFD.PGE[order(SED.SD.PGE_SED.HFD.PGE$padj),]
SED.SD.PGE_SED.HFD.PGE
summary(SED.SD.PGE_SED.HFD.PGE)
sum(SED.SD.PGE_SED.HFD.PGE$padj < 0.1, na.rm = TRUE)
sum(SED.SD.PGE_SED.HFD.PGE$padj < 0.05, na.rm = TRUE)
write.csv(as.data.frame(SED.SD.PGE_SED.HFD.PGE), file =
"/home/afb/AL3_data/AL3_SED.SD.PGE_SED.HFD.PGE.csv")

#### 17)  PA.SD.SAL vs PA.SD.PGE ######
PA.SD.SAL_PA.SD.PGE <- results(ddsIF_3, contrast = c("group", "PASDSAL", "PASDPGE" ))
PA.SD.SAL_PA.SD.PGE <- PA.SD.SAL_PA.SD.PGE[order(PA.SD.SAL_PA.SD.PGE$padj),]
PA.SD.SAL_PA.SD.PGE
summary(PA.SD.SAL_PA.SD.PGE)
sum(PA.SD.SAL_PA.SD.PGE$padj < 0.1, na.rm = TRUE)
sum(PA.SD.SAL_PA.SD.PGE$padj < 0.05, na.rm = TRUE)
write.csv(as.data.frame(PA.SD.SAL_PA.SD.PGE), file =
"/home/afb/AL3_data/AL3_PA.SD.SAL_PA.SD.PGE.csv")

#### 18)  PA.HFD.SAL vs PA.HFD.PGE ######
PA.HFD.SAL_PA.HFD.PGE <- results(ddsIF_3, contrast = c("group", "PAHFDSAL", "PAHFDPGE" ))
PA.HFD.SAL_PA.HFD.PGE <- PA.HFD.SAL_PA.HFD.PGE[order(PA.HFD.SAL_PA.HFD.PGE$padj),]
```

```
PA.HFD.SAL_PA.HFD.PGE
summary(PA.HFD.SAL_PA.HFD.PGE)
sum(PA.HFD.SAL_PA.HFD.PGE$padj < 0.1, na.rm = TRUE)
sum(PA.HFD.SAL_PA.HFD.PGE$padj < 0.05, na.rm = TRUE)
write.csv(as.data.frame(PA.HFD.SAL_PA.HFD.PGE), file =
"/home/afb/AL3_data/AL3_PA.HFD.SAL_PA.HFD.PGE.csv")

#### 19)  PA.SD.SAL vs PA.HFD.SAL ######
PA.SD.SAL_PA.HFD.SAL <- results(ddsIF_3, contrast = c("group", "PASDSAL", "PAHFDSAL" ))
PA.SD.SAL_PA.HFD.SAL <- PA.SD.SAL_PA.HFD.SAL[order(PA.SD.SAL_PA.HFD.SAL$padj),]
PA.SD.SAL_PA.HFD.SAL
summary(PA.SD.SAL_PA.HFD.SAL)
sum(PA.SD.SAL_PA.HFD.SAL$padj < 0.1, na.rm = TRUE)
sum(PA.SD.SAL_PA.HFD.SAL$padj < 0.05, na.rm = TRUE)
write.csv(as.data.frame(PA.SD.SAL_PA.HFD.SAL), file =
"/home/afb/AL3_data/AL3_PA.SD.SAL_PA.HFD.SAL.csv")

#### 20)  PA.SD.PGE vs PA.HFD.PGE ######
PA.SD.PGE_PA.HFD.PGE <- results(ddsIF_3, contrast = c("group", "PASDPGE", "PAHFDPGE" ))
PA.SD.PGE_PA.HFD.PGE <- PA.SD.PGE_PA.HFD.PGE[order(PA.SD.PGE_PA.HFD.PGE$padj),]
PA.SD.PGE_PA.HFD.PGE
summary(PA.SD.PGE_PA.HFD.PGE)
sum(PA.SD.PGE_PA.HFD.PGE$padj < 0.1, na.rm = TRUE)
sum(PA.SD.PGE_PA.HFD.PGE$padj < 0.05, na.rm = TRUE)
write.csv(as.data.frame(PA.SD.PGE_PA.HFD.PGE), file =
"/home/afb/AL3_data/AL3_PA.SD.PGE_PA.HFD.PGE.csv")

#### 21)  SED.HFD.SAL vs PA.HFD.SAL ######
SED.HFD.SAL_PA.HFD.SAL <- results(ddsIF_3, contrast = c("group", "SEDHFDSAL", "PAHFDSAL" ))
SED.HFD.SAL_PA.HFD.SAL <- SED.HFD.SAL_PA.HFD.SAL[order(SED.HFD.SAL_PA.HFD.SAL$padj),]
SED.HFD.SAL_PA.HFD.SAL
summary(SED.HFD.SAL_PA.HFD.SAL)
sum(SED.HFD.SAL_PA.HFD.SAL$padj < 0.1, na.rm = TRUE)
sum(SED.HFD.SAL_PA.HFD.SAL$padj < 0.05, na.rm = TRUE)
write.csv(as.data.frame(SED.HFD.SAL_PA.HFD.SAL), file =
"/home/afb/AL3_data/AL3_SED.HFD.SAL_PA.HFD.SAL.csv")

#### 22)  SED.HFD.PGE vs PA.HFD.PGE ######
SED.HFD.PGE_PA.HFD.PGE <- results(ddsIF_3, contrast = c("group", "SEDHFDPGE", "PAHFDPGE" ))
SED.HFD.PGE_PA.HFD.PGE <- SED.HFD.PGE_PA.HFD.PGE[order(SED.HFD.PGE_PA.HFD.PGE$padj),]
SED.HFD.PGE_PA.HFD.PGE
summary(SED.HFD.PGE_PA.HFD.PGE)
sum(SED.HFD.PGE_PA.HFD.PGE$padj < 0.1, na.rm = TRUE)
sum(SED.HFD.PGE_PA.HFD.PGE$padj < 0.05, na.rm = TRUE)
mcols(ddsIF_3, use.names = TRUE)[1:4,1:4] # acesso aos valores de dispersão, coeficientes e SE
write.csv(as.data.frame(SED.HFD.PGE_PA.HFD.PGE), file =
"/home/afb/AL3_data/AL3_SED.HFD.PGE_PA.HFD.PGE.csv")

#--------------# Volcano Plot #---------------------------#

tmp <- read.csv("~/AL3_data/Volcano_Nam/22_AL3_SED.HFD.PGE_PA.HFD.PGE.csv", stringsAsFactors=F)

rownames(tmp) <- tmp$X
tmp$plog <- -log10(tmp$padj)
tmp$status <- "Non-significant"
tmp$status[tmp$plog > -log10(0.05) & tmp$log2FoldChange >  0] <- "Up-regulated"
tmp$status[tmp$plog > -log10(0.05) & tmp$log2FoldChange < 0] <- "Down-regulated"
tmp$label <- 0
#hot <- c("ENSMUSG00000041559", "ENSMUSG00000035456", "ENSMUSG00000081810",
"ENSMUSG00000024175")
tmp[tmp$X %in% hot, ]$label <- 1
```

```
ggplot(tmp, aes(x=log2FoldChange, y=plog, color=status)) +
  geom_point(alpha=0.4, size=2, stroke=0.5) +
  scale_color_manual(values=c("red", "grey0", "blue1")) +
  geom_hline(yintercept=-log10(0.05), linetype="dashed", color="black") +
  #geom_vline(xintercept=c(-1, 1), linetype="dashed", color="black") +
  coord_cartesian(xlim=c(-2.5, 3), ylim=c(0, 3)) +
  labs(title="SED.HFD.PGE vs PA.HFD.PGE",
       x=expression('log'[2]*' Fold Change'),
       y=expression('-log'[10]*'(adj p-value)'),
       subtitle=paste(nrow(tmp), "Genes")) +
  geom_text_repel(
    data=subset(tmp, label==1), aes(label=X),
    size = 2,
    color="black",
    force = 7000,
    segment.size  = 0.3,
    segment.color = "black",
    point.padding = 0.3,
    box.padding = 1,
    direction     = "both",
    fill = "white",
    show.legend = FALSE) +
    theme_classic() +
    theme(axis.line = element_line(colour = "black", size = 0.8, linetype = "solid")) +
  theme(axis.text.x = element_text(face="bold", color="black", size=14, angle=0),
      axis.text.y = element_text(face="bold", color="black", size=14, angle=0)) +
  theme(axis.title = element_text(face = "bold", color = "black", size = 16)) +
  theme(legend.text = element_text(face = "plain", color = "black", size = 16))
#--------------# Volcano Plot #---------------------------#

#### 23)  SED.HFD.SAL vs PA.HFD.PGE ######
SED.HFD.SAL_PA.HFD.PGE <- results(ddsIF_3, contrast = c("group", "SEDHFDSAL", "PAHFDPGE" ))
SED.HFD.SAL_PA.HFD.PGE <- SED.HFD.SAL_PA.HFD.PGE[order(SED.HFD.SAL_PA.HFD.PGE$padj),]
SED.HFD.SAL_PA.HFD.PGE
summary(SED.HFD.SAL_PA.HFD.PGE)
sum(SED.HFD.SAL_PA.HFD.PGE$padj < 0.1, na.rm = TRUE)
sum(SED.HFD.SAL_PA.HFD.PGE$padj < 0.05, na.rm = TRUE)
write.csv(as.data.frame(SED.HFD.SAL_PA.HFD.PGE), file =
"/home/afb/AL3_data/AL3_SED.HFD.SAL_PA.HFD.PGE.csv")

#### 24)  SED.HFD.PGE vs PA.HFD.SAL ######
SED.HFD.PGE_PA.HFD.SAL <- results(ddsIF_3, contrast = c("group", "SEDHFDPGE", "PAHFDSAL" ))
SED.HFD.PGE_PA.HFD.SAL <- SED.HFD.PGE_PA.HFD.SAL[order(SED.HFD.PGE_PA.HFD.SAL$padj),]
SED.HFD.PGE_PA.HFD.SAL
summary(SED.HFD.PGE_PA.HFD.SAL)
sum(SED.HFD.PGE_PA.HFD.SAL$padj < 0.1, na.rm = TRUE)
sum(SED.HFD.PGE_PA.HFD.SAL$padj < 0.05, na.rm = TRUE)
write.csv(as.data.frame(SED.HFD.PGE_PA.HFD.SAL), file =
"/home/afb/AL3_data/AL3_SED.HFD.PGE_PA.HFD.SAL.csv")

#### 25)  SED.SD.SAL vs SED.HFD.PGE ######
SED.SD.SAL_SED.HFD.PGE <- results(ddsIF_3, contrast = c("group", "SEDSDSAL", "SEDHFDPGE" ))
SED.SD.SAL_SED.HFD.PGE <- SED.SD.SAL_SED.HFD.PGE[order(SED.SD.SAL_SED.HFD.PGE$padj),]
SED.SD.SAL_SED.HFD.PGE
summary(SED.SD.SAL_SED.HFD.PGE)
sum(SED.SD.SAL_SED.HFD.PGE$padj < 0.1, na.rm = TRUE)
sum(SED.SD.SAL_SED.HFD.PGE$padj < 0.05, na.rm = TRUE)
write.csv(as.data.frame(SED.SD.SAL_SED.HFD.PGE), file =
"/home/afb/AL3_data/AL3_SED.SD.SAL_SED.HFD.PGE.csv")

#### 26)  SED.SD.PGE vs SED.HFD.SAL ######
SED.SD.PGE_SED.HFD.SAL <- results(ddsIF_3, contrast = c("group", "SEDSDPGE", "SEDHFDSAL" ))
```

```
SED.SD.PGE_SED.HFD.SAL <- SED.SD.PGE_SED.HFD.SAL[order(SED.SD.PGE_SED.HFD.SAL$padj),]
SED.SD.PGE_SED.HFD.SAL
summary(SED.SD.PGE_SED.HFD.SAL)
sum(SED.SD.PGE_SED.HFD.SAL$padj < 0.1, na.rm = TRUE)
sum(SED.SD.PGE_SED.HFD.SAL$padj < 0.05, na.rm = TRUE)
write.csv(as.data.frame(SED.SD.PGE_SED.HFD.SAL), file =
"/home/afb/AL3_data/AL3_SED.SD.PGE_SED.HFD.SAL.csv")

#### 27)  PA.SD.SAL vs PA.HFD.PGE ######
PA.SD.SAL_PA.HFD.PGE <- results(ddsIF_3, contrast = c("group", "PASDSAL", "PAHFDPGE" ))
PA.SD.SAL_PA.HFD.PGE <- PA.SD.SAL_PA.HFD.PGE[order(PA.SD.SAL_PA.HFD.PGE$padj),]
PA.SD.SAL_PA.HFD.PGE
summary(PA.SD.SAL_PA.HFD.PGE)
sum(PA.SD.SAL_PA.HFD.PGE$padj < 0.1, na.rm = TRUE)
sum(PA.SD.SAL_PA.HFD.PGE$padj < 0.05, na.rm = TRUE)
write.csv(as.data.frame(PA.SD.SAL_PA.HFD.PGE), file =
"/home/afb/AL3_data/AL3_PA.SD.SAL_PA.HFD.PGE.csv")

#### 28)  PA.SD.PGE vs PA.HFD.SAL ######
PA.SD.PGE_PA.HFD.SAL <- results(ddsIF_3, contrast = c("group", "PASDPGE", "PAHFDSAL" ))
PA.SD.PGE_PA.HFD.SAL <- PA.SD.PGE_PA.HFD.SAL[order(PA.SD.PGE_PA.HFD.SAL$padj),]
PA.SD.PGE_PA.HFD.SAL
summary(PA.SD.PGE_PA.HFD.SAL)
sum(PA.SD.PGE_PA.HFD.SAL$padj < 0.1, na.rm = TRUE)
sum(PA.SD.PGE_PA.HFD.SAL$padj < 0.05, na.rm = TRUE)
write.csv(as.data.frame(PA.SD.PGE_PA.HFD.SAL), file =
"/home/afb/AL3_data/AL3_PA.SD.PGE_PA.HFD.SAL.csv")

#-----------------------------------#-------------------------------------------#-------------------------------------------#
```
